# ASC4OPT: asciminib treatment optimization study in patients with chronic myeloid leukemia in chronic phase previously treated with two or more tyrosine kinase inhibitors

**DOI:** 10.1038/s41375-026-02965-8

**Published:** 2026-04-23

**Authors:** Andreas Hochhaus, Philipp le Coutre, Dragana Milojkovic, Dennis Dong Hwan Kim, Soo Min Lim, Carolina Pavlovsky, Thanh Nguyen, Franck Emmanuel Nicolini, Beatriz Moiraghi, Sebastian Grosicki, Chi Dung Phu, Gabriel Etienne, Fernando Marco de Lucas, Rosa Maria Ayala Diaz, Massimo Breccia, Charles Chuah, Roberto Abi Rached, Himanshu Pokhriyal, Aswin IC, Peter Schuld, Virginia Pilipovic, Franz Alisch, Carla Maria Boquimpani

**Affiliations:** 1 Klinik für Innere Medizin II, Hematology/Oncology, Universitätsklinikum Jena and Comprehensive Cancer Center Central Germany, Campus Jena, Jena, Germany; 2 Department of Oncology and Hematology, Charité-Universitätsmedizin Berlin, Berlin, Germany; 3 Centre for Haematology, Department of Immunology and Inflammation, Imperial College London, and Department of Clinical Haematology, Imperial College Healthcare NHS Trust, London, UK; 4 Princess Margaret Cancer Centre, University Health Network, University of Toronto, Toronto, ON Canada; 5 Sultanah Aminah Hospital, Johor Bahru, Malaysia; 6 FUNDALEU, Buenos Aires, Argentina; 7 National Institute of Hematology and Blood Transfusion, Hanoi, Vietnam; 8 Department of Hematology, Centre Léon Bérard, and INSERM U1052, CRCL, Lyon, France; 9 Hospital Jose Maria Ramos Mejia, Buenos Aires, Argentina; 10 Department of Hematology and Cancer Prevention, Faculty of Health Sciences in Bytom, Medical University of Silesia, Katowice, Poland; 11 Blood Transfusion Hematology Hospital, Ho Chi Minh City, Vietnam; 12 Hematology Department, Institut Bergonié, Bordeaux, France; 13 Servicio de Hematología, Hospital Universitario Basurto, Osakidetza, Bilbao, Spain; 14 Haematological Malignancies Clinical Research Unit, Hospital 12 de Octubre Universidad Complutense, CNIO, CIBERONC and Spanish National Cancer Research Center (CNIO), Madrid, Spain; 15 Hematology, Department of Translational and Precision Medicine, Sapienza University of Rome, Rome, Italy; 16 Singapore General Hospital, Singhealth Duke-NUS Medical School, Singapore, Singapore; 17 Novartis Pharma AG, Basel, Switzerland; 18 Novartis Healthcare Pvt. Ltd., Salarpuria-Sattva Knowledge City, Hyderabad, India; 19 HEMORIO, State Institute of Hematology Arthur de Siqueira Cavalcanti, Rio de Janeiro, Brazil

**Keywords:** Drug development, Molecularly targeted therapy

## Abstract

The ASC4OPT non-comparative phase 3b study (NCT04948333) evaluates asciminib once daily (QD) or twice daily (BID) in chronic myeloid leukemia in chronic phase (CML-CP) treated with ≥2 tyrosine kinase inhibitors (TKIs). This study enrolled 169 patients not in major molecular response (MMR), with unsatisfactory response (intolerant, warning or failure) as defined by European LeukemiaNet (ELN) 2020 criteria. Patients intolerant to their most recent TKI and in MMR at baseline (*n* = 30) were also enrolled. The primary endpoint was the MMR rate at Week 48 for patients not in MMR at baseline. Results showed an overall MMR rate of 39.4% at Week 48 (40 mg BID, 43.4%; 80 mg QD, 35.4%) and 43.6% at Week 96 (40 mg BID, 45.8%; 80 mg QD, 41.5%) in patients not in MMR at baseline. Among 40 patients who had their asciminib dose escalated to 200 mg QD, 17.5% were in MMR at Week 96. Most patients in MMR at baseline remained in MMR at 48 and 96 weeks (93.3% and 86.7%, respectively). Safety for both dosing regimens was consistent with that of previous studies. Findings support asciminib as a potential standard of care for patients with CML-CP who have not responded optimally to prior TKI therapy.

## Introduction

Asciminib is the first BCR::ABL1 inhibitor that works by Specifically Targeting the ABL Myristoyl Pocket (STAMP) [1–3]. It is approved in several countries, including those of the European Union and the United States (US), for the treatment of newly diagnosed or previously treated adult patients with Philadelphia chromosome-positive (Ph + ) chronic myeloid leukemia in chronic phase (CML-CP) at a dose of 40 mg twice daily (BID) or 80 mg once daily (QD) for patients not harboring the T315I mutation [4–7]. For patients with the T315I mutation, the recommended dose is 200 mg BID [4].

Asciminib was initially approved for patients treated with ≥2 tyrosine kinase inhibitors (TKIs) and not harboring the T315I mutation based on the results of the pivotal phase 3 ASCEMBL study, which showed superior efficacy and a favorable safety and tolerability profile for asciminib 40 mg BID compared with bosutinib 500 mg QD [8, 9]. Criteria for inclusion in the ASCEMBL study were treatment failure as defined in the 2013 European LeukemiaNet (ELN) recommendations or intolerance to the most recent TKI [8, 10]. Since the design of the ASCEMBL study, ELN updated its definition of warning response, adding further criteria to describe the patient population that is not resistant to treatment but does not respond optimally either [11]. Although patients with warning response constitute a heterogeneous population, clinical studies have shown that this level of response can be associated with poorer outcomes compared with optimally responding patients [12–14].

Some patients may achieve treatment milestones on their current TKI but may experience intolerance to an extent that significantly impacts their quality of life (QoL), for example, persistent low-grade adverse events (AEs) [15]. Patients who switch TKI treatment due to intolerance can achieve optimal outcomes if they stay on their new treatment [16–18]; however, not all patients respond optimally to, or have improved tolerability on, new treatments [11].

Asciminib is administered under fasting conditions and, therefore, a twice-daily dosing regimen may be challenging for patients to accommodate meal scheduling. The prolonged nature of CML treatment likely exacerbates this situation and may lead to reduced compliance with treatment. Pharmacokinetic analyses in patients enrolled in the dose-finding phase 1 asciminib study showed that, at doses of 40 mg BID or 80 mg QD, asciminib trough blood concentrations exceeded the preclinical 90% inhibitory concentration for phosphorylated signal transducer and activator of transcription 5 (pSTAT5) inhibition, a downstream marker of BCR::ABL1 inhibition [19]. Furthermore, asciminib population pharmacokinetics and exposure-response analyses showed that asciminib at 40 mg BID and 80 mg QD had similar exposure and effects on *BCR::ABL1*^IS^ percentages over time [20, 21]. These results led to the approval of the 80 mg QD dosing regimen in the US and several other countries. An 80 mg QD dosing schedule is likely more convenient for patients and may improve treatment adherence, potentially resulting in better outcomes. The 80 mg QD dosing schedule has already demonstrated superior efficacy and favorable safety and tolerability versus all current standard-of-care TKIs in newly diagnosed patients enrolled in the ASC4FIRST study [22].

The ASC4OPT study explored several aspects of asciminib treatment optimization, including investigating two different dosing schedules for asciminib, re-evaluating the efficacy of asciminib in an expanded third-line or later (3 L + ) population using the ELN 2020 treatment recommendations for warning and failure criteria [11], assessing the potential benefit of a dose escalation of asciminib to 200 mg QD for patients not in major molecular response (MMR) at ≥48 weeks, and exploring whether asciminib treatment could maintain levels of response with a favorable safety profile in patients already in MMR at study entry. We report here the results of the ASC4OPT study up to Week 96, showing that asciminib is highly efficacious and has a favorable safety profile at both 40 mg BID and 80 mg QD regimens in a broader population of patients, including those with warning response and those already in MMR at baseline.

## Methods

### Study design and patients

ASC4OPT (ClinicalTrials.gov NCT04948333, first submitted 28 June 2021) is an international, multicenter, open-label, non-comparative phase 3b study conducted in adults with CML-CP previously treated with ≥2 TKIs (imatinib, nilotinib, dasatinib, bosutinib, radotinib, or ponatinib). The study protocol and statistical analysis plan can be accessed in ClinicalTrials.gov. The sponsor (Novartis) and the trial steering committee collaboratively designed the trial and interpreted the data. Eligible patients for the main trial cohort were in treatment failure or warning categories according to ELN 2020 criteria [11], or were intolerant to their most recent TKI and not in MMR at baseline (see Supplementary Methods for details). Patients harboring the T315I mutation, having a history of accelerated phase or blast phase, or having undergone or planning to undergo hematopoietic stem cell transplant were excluded. An exploratory group of patients intolerant to their most recent TKI and in MMR at baseline (exploratory cohort) was also enrolled; assessments for these patients were conducted as for the main cohort, but data were not included in the primary endpoint analysis. Analysis sets are presented in Supplementary Methods.

All patients were randomized 1:1 by interactive response technology (IRT) to receive either asciminib 40 mg BID or 80 mg QD under fasting conditions for a maximum of 144 weeks (Supplementary Fig. 1).

For patients in the main cohort not achieving MMR at Week 48 or losing response after Week 48 and up to Week 108, the asciminib dose could be escalated to 200 mg QD if the investigator considered this to be beneficial. This dose of asciminib was expected to lead to a marked increase in exposure, which may in turn lead to improved efficacy; at the same time, this dose remained within the known safety profile of asciminib, which has been assessed up to doses of 200 mg BID [18]. For dose escalation to be considered, there must have been no Grade ≥3 or persistent Grade 2 toxicity on therapy considered to be possibly related to asciminib and unresponsive to optimal management.

### Study endpoints

The primary endpoint was MMR rate at Week 48 for patients in the main cohort; patients who discontinued earlier for any reason were considered non-responders at Week 48. Secondary endpoints for this population included MMR rate at Week 96, time to and duration of MMR, rates of deep molecular response (MR^4^ [*BCR::ABL1*^IS^ ≤ 0.01%] and MR^4.5^ [*BCR::ABL1*^IS^ ≤ 0.0032%]), *BCR::ABL1*^IS^ ≤ 1%, and complete cytogenetic response (CCyR) at and by scheduled time points, patient-reported outcomes (PROs), and safety and tolerability. For patients in the exploratory cohort, MMR rate at Week 48 was assessed separately as a secondary endpoint; other secondary endpoints for this patient group included MMR rate at Week 96, PROs, and safety.

### Study assessments

AEs were categorized using the Medical Dictionary for Regulatory Activities (MedDRA) version 27.1 and assigned grades according to the Common Terminology Criteria for Adverse Events (CTCAE) version 5.0. Molecular response was evaluated by real-time, quantitative, reverse-transcriptase polymerase chain reaction (RT-qPCR), with results presented as the ratio of *BCR::ABL1* to *ABL1* on the International Scale (IS) [23]. Bone marrow assessments were conducted in the main cohort to evaluate CCyR rates at Week 48; patients without an available bone marrow assessment who were in MMR at Week 48 were considered as having achieved CCyR. *BCR::ABL1* mutation analysis was conducted via next-generation sequencing (NGS) at baseline; post-baseline mutation analysis included data from NGS and Sanger sequencing. All molecular assessments were performed in a centralized laboratory (Molecular MD, Portland, OR, USA). PROs were assessed by the MD Anderson Symptom Inventory—Chronic Myelogenous Leukemia (MDASI-CML) questionnaire.

### Statistical analysis

Demographic and other baseline data, including disease characteristics, were listed and summarized descriptively. Categorical data were presented as frequencies and percentages; for continuous data, median, minimum, and maximum values were presented. For efficacy endpoints, the rate and the associated 95% two-sided confidence interval (CI) based on the Clopper-Pearson method were presented. The cumulative incidence of MMR was calculated considering discontinuation from study treatment for any reason and without prior achievement of MMR as a competing risk.

### Ethics

The study was designed collaboratively by the sponsor (Novartis Pharma AG) and lead study investigators. The protocol was approved by the independent ethics committee, or institutional review board, at each participating site and the study was conducted in accordance with Good Clinical Practice guidelines and the Declaration of Helsinki. All patients provided written informed consent.

## Results

### Patients

**Fig. 1:**
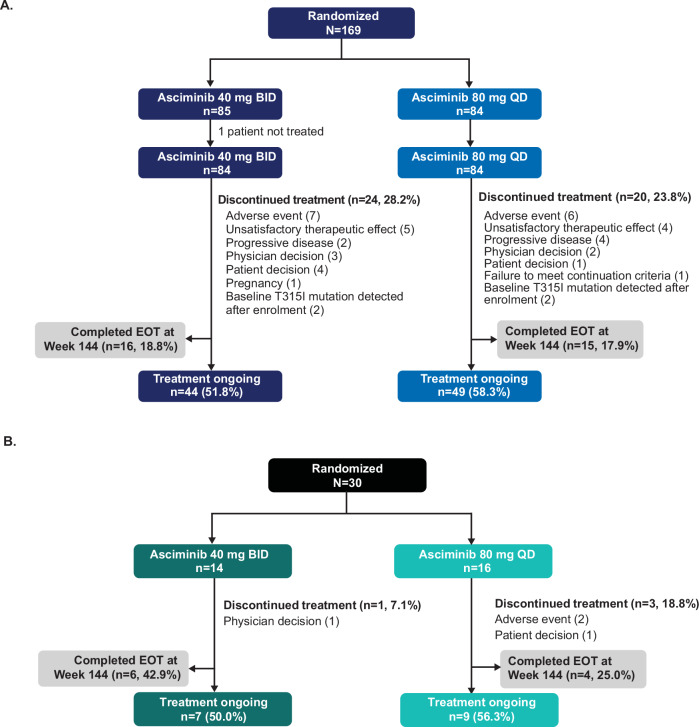
Patient disposition. **A** Main cohort; **B** Exploratory cohort. BID, twice daily; EOT end of treatment, QD once daily.

Overall, 169 patients who were not in MMR at baseline were enrolled in the main cohort between November 2021 and March 2023 and randomized to asciminib 40 mg BID (*n* = 85) or 80 mg QD (*n* = 84) (Fig. 1A). One patient in the 40 mg BID arm was not treated. At data cutoff (11 February 2025), after a median follow-up of 124.1 weeks (28.6 months), 31 patients (18.3%) had completed the end of treatment (EOT) visit at Week 144, and treatment was ongoing for 93 patients (55.0%); most patients who discontinued asciminib (17/44) did so before Week 24.

A total of 40 patients in the main cohort (16 in the 40 mg BID arm and 24 in the 80 mg QD arm) had their asciminib dose escalated to 200 mg QD, all except one due to not achieving MMR at Week 48. By data cutoff, 11 of these patients had completed the EOT visit at Week 144, and 24 were receiving treatment with the higher asciminib dose. Among the patients who discontinued treatment (*n* = 5), progressive disease was the most common reason (*n* = 2).

Patients were heavily pretreated, and most had discontinued their previous TKI due to resistance (Table 1 and Supplementary Fig. 2). Although the study arms were balanced in terms of age and race, there were differences across treatment arms in other categories (sex, ethnicity, number of previous TKI treatment lines, reason for discontinuation of last TKI, response level at baseline). Patient demographics and disease characteristics for the patients who had their asciminib dose escalated to 200 mg QD are presented in Supplementary Table 1.

**Table 1:** Patient demographics and disease characteristics (main and exploratory cohorts).

	Main cohort			Exploratory cohort		
	Asciminib 40 mg BID *n* = 85	Asciminib 80 mg QD *n* = 84	All patients *N* = 169	Asciminib 40 mg BID *n* = 14	Asciminib 80 mg QD *n* = 16	All patients *N* = 30
Median age, years (range)	54.0 (24‒86)	56.0 (18‒84)	55.0 (18‒86)	56.5 (37‒81)	63.0 (32‒77)	59.0 (32‒81)
Male, *n* (%)	48 (56.5)	57 (67.9)	105 (62.1)	8 (57.1)	10 (62.5)	18 (60.0)
Race, *n* (%)						
White	58 (68.2)	60 (71.4)	118 (69.8)	12 (85.7)	14 (87.5)	26 (86.7)
Black or African American	4 (4.7)	2 (2.4)	6 (3.6)	–	–	–
Asian	21 (24.7)	21 (25.0)	42 (24.9)	2 (14.3)	2 (12.5)	4 (13.3)
Multiple	1 (1.2)	0	1 (0.6)	–	–	–
Unknown	1 (1.2)	1 (1.2)	2 (1.2)	–	–	–
Ethnicity, *n* (%)						
Hispanic or Latino	12 (14.1)	17 (20.2)	29 (17.2)	1 (7.1)	2 (12.5)	3 (10.0)
Not Hispanic or Latino	63 (74.1)	58 (69.0)	121 (71.6)	12 (85.7)	13 (81.3)	25 (83.3)
Not Reported	7 (8.2)	6 (7.1)	13 (7.7)	1 (7.1)	0	1 (3.3)
Unknown	3 (3.5)	3 (3.6)	6 (3.6)	0	1 (6.3)	1 (3.3)
Median time since initial diagnosis of CML, years (range)	3.8 (0.2‒29.6)	3.7 (0.6‒28.1)	3.7 (0.2‒29.6)	4.4 (1.7‒21.2)	5.2 (1.3‒18.0)	4.5 (1.3‒21.2)
Number of lines of prior TKI therapy, *n* (%)						
2	45 (52.9)	39 (46.4)	84 (49.7)	6 (42.9)	8 (50.0)	14 (46.7)
3	23 (27.1)	27 (32.1)	50 (29.6)	3 (21.4)	3 (18.8)	6 (20.0)
4	9 (10.6)	17 (20.2)	26 (15.4)	3 (21.4)	3 (18.8)	6 (20.0)
≥5	7 (8.2)	1 (1.2)	8 (4.7)	2 (14.3)	2 (12.5)	4 (13.3)
Reason to discontinue last TKI, *n* (%)						
Resistance	43 (50.6)	46 (54.8)	89 (52.7)	1 (7.1)	1 (6.3)	2 (6.7)
Intolerance	19 (22.4)	20 (23.8)	39 (23.1)	10 (71.4)	11 (68.8)	21 (70.0)
Others^a^	22 (25.9)	18 (21.4)	40 (23.7)	3 (21.4)	4 (25.0)	7 (23.3)
Cytogenetic response^b^, *n* (%)						
CCyR	27 (31.8)	29 (34.5)	56 (33.1)			
PCyR	18 (21.2)	9 (10.7)	27 (16.0)			
*BCR::ABL1*^IS^ > 10%, *n* (%)	36 (42.4)	31 (36.9)	67 (39.6)			

*BID* twice daily, *CCyR* complete cytogenetic response, *CML* chronic myeloid leukemia, *IS* international scale, *MMR* major molecular response, *PCyR* partial cytogenetic response, *QD* once daily, *TKI* tyrosine kinase inhibitor.

^a^Other reasons cited included “Patient was in remission and therapy was paused”, “Unknown”, “Completed prescribed regimen”, “Medication no longer required”, “Change of TKI”, “Indisposition, planned change of TKI”, “Medical decision”, “MMR failure”, “Started on very low dose while awaiting enrollment on CABL001A2302 for asciminib”, “Started on study drug”, “Anemia Grade 3, muscle stiffness (Grade 3), nausea, diarrhea”. ^b^ Other categories included minor, minimal, none and missing.

The exploratory cohort of 30 patients already in MMR at baseline was randomized to asciminib 40 mg BID (*n* = 14) or 80 mg QD (*n* = 16). Patients in this cohort were similar to patients in the main cohort regarding age and sex; however, there was a larger proportion of patients who had received ≥5 previous treatment lines in this group compared with patients not in MMR at baseline (Table 1). Median time from initial diagnosis of CML was 4.5 years (range 1.3─21.2). At data cutoff, 10 of these patients (33.3%) had completed the EOT visit at Week 144 and treatment was ongoing for 16 patients (53.3%) (Fig. 1B). One patient in the 40 mg BID arm (physician decision) and three patients in the 80 mg QD arm discontinued treatment (AEs, *n* = 2; patient decision, *n* = 1).

### Efficacy: primary endpoint

The MMR rate at Week 48 for patients in the main cohort was 39.4% (95% CI: 31.9, 47.3); 43.4% (95% CI: 32.5, 54.7) in the 40 mg BID arm and 35.4% (95% CI: 25.1, 46.7) in the 80 mg QD arm (Table 2). Response rates over time (at Week 48 and Week 96) are detailed in Table 3. MMR rates at and by scheduled time points are presented in Supplementary Tables 2 and 3. Of note, the T315I mutation was detected in four patients after treatment start; although the sample for mutation testing was collected before patients received asciminib, treatment had already started by the time the results were available; these patients were excluded from efficacy analyses.

**Table 2:** MMR at Week 48 (main cohort).

	Asciminib 40 mg BID *n* = 83		Asciminib 80 mg QD *n* = 82		All patients *N* = 165	
	*n* (%)	95% CI	*n* (%)	95% CI	*n* (%)	95% CI
At Week 48	36 (43.4)	(32.53, 54.71)	29 (35.4)	(25.12, 46.70)	65 (39.4)	(31.89, 47.29)

*BID* twice daily, *CI* confidence interval, *MMR* major molecular response, *QD* once daily, *RT-qPCR* reverse transcription quantitative polymerase chain reaction.

Four patients with a baseline T315I mutation detected after treatment start were excluded from these analyses. Patients without RT-qPCR assessment at 48 weeks were considered as non-responders at Week 48, unless both 36- and 60-week RT-qPCR assessments indicated that the patient was in MMR.

**Table 3:** Response rates over time (main cohort).

	MMR			MR^4^			MR^4.5^			*BCR::ABL1*^IS^ ≤ 1%		
*n* (%)	Asciminib 40 mg BID *n* = 83	Asciminib 80 mg QD *n* = 82	All patients *N* = 165	Asciminib 40 mg BID *n* = 83	Asciminib 80 mg QD *n* = 82	All patients N = 165	Asciminib 40 mg BID *n* = 83	Asciminib 80 mg QD *n* = 82	All patients *N* = 165	Asciminib 40 mg BID *n* = 83	Asciminib 80 mg QD *n* = 82	All patients *N* = 165
At Week 48	36 (43.4)	29 (35.4)	65 (39.4)	17 (20.5)	11 (13.4)	28 (17.0)	10 (12.0)	7 (8.5)	17 (10.3)	55 (66.3)	50 (61.0)	105 (63.6)
At Week 96	38 (45.8)	34 (41.5)	72 (43.6)	18 (21.7)	10 (12.2)	28 (17.0)	11 (13.3)	7 (8.5)	18 (10.9)	52 (62.7)	55 (67.1)	107 (64.8)

*BID* twice daily, *IS* international scale, *MMR* major molecular response, *MR* molecular response, *QD* once daily, *RT-qPCR* reverse transcription quantitative polymerase chain reaction.

Four patients with the T315I mutation detected after treatment start were excluded from these analyses. For MMR, patients without RT-qPCR assessment at 48 weeks were considered as non-responders at Week 48, unless both 36- and 60-week RT-qPCR assessments indicated that the patient was in MMR. Patients without RT-qPCR assessment at 96 weeks were considered as non-responders at Week 96, unless both 84- and 108-week RT-qPCR assessments indicated that the patient was in MMR. For other response types, patients without RT-qPCR assessment at a time point were considered as non-responders at that time point.

The dosing arm populations were not balanced, with the 40 mg BID arm including a larger proportion of female patients and patients aged <65 years, as well as a lower proportion of patients who had discontinued their previous TKI due to resistance or who had received three or more previous TKIs (Supplementary Table 4). Given this imbalance, an ad-hoc statistical analysis using propensity weighting scores was conducted to investigate the numerical differences in MMR rates. Differences were not significant for MMR rates at Week 48 for patients treated with asciminib 40 mg BID or 80 mg QD (ad-hoc nominal *p* value, *p* = 0.763; Supplementary Table 5). Since MMR rates in ASC4OPT were numerically higher than those reported in the ASCEMBL study (43.4% vs 29.0% for asciminib 40 mg BID at Week 48) [8], a separate propensity weighting score analysis was conducted to investigate the differences between the MMR rates. As shown in Supplementary Table 5, no significant differences were observed between MMR rates in ASC4OPT and ASCEMBL.

### Efficacy: secondary endpoints

**Fig. 2:**
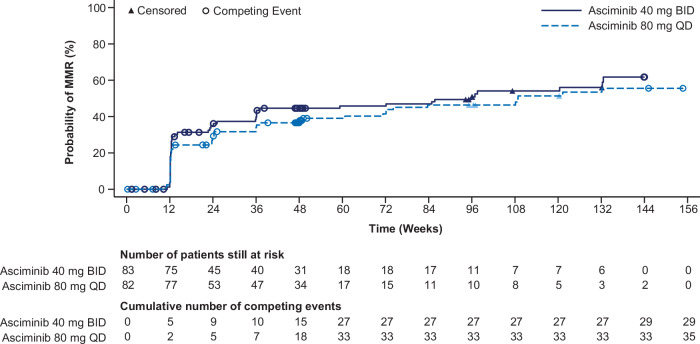
Cumulative response plot of time to first MMR (main cohort). Patients without RT-qPCR assessment at a certain time point are considered as non-responders at that time point. BID, twice daily; MMR, major molecular response; QD, once daily; RT-qPCR, reverse transcription quantitative polymerase chain reaction.

The estimated median time to first MMR among those who achieved it was 22.7 weeks (13.1 weeks and 23.9 weeks for patients on asciminib 40 mg BID and 80 mg QD, respectively). MMR rates continued to increase throughout the study, reaching similar levels in both arms at Week 96 (45.8% and 41.5% for 40 mg BID and 80 mg QD, respectively) (Table 3). Figure 2 shows cumulative MMR rates; the median duration of MMR was not estimable for either dosing regimen (Supplementary Fig. 3).

MR^4^ and MR^4.5^ rates at Week 48 were 17.0% and 10.3%, respectively (20.5% and 12.0% for patients on 40 mg BID, and 13.4% and 8.5% for patients on 80 mg QD, respectively); at Week 96, the corresponding values were 17.0% and 10.9%, respectively. (Table 3, and Supplementary Tables 6 and 8); data by scheduled time points are presented in Supplementary Tables 7 and 9).

At Week 48 and Week 96, *BCR::ABL1*^IS^ ≤ 1% was achieved in 63.6% (66.3% on 40 mg BID and 61.0% on 80 mg QD) and 64.8% (62.7% on 40 mg BID and 67.1% on 80 mg QD) of patients, respectively (Table 3 and Supplementary Tables 10 and 11). At Week 48, the CCyR rate was 58.2% for all patients (60.2% and 56.1% for patients on asciminib 40 mg BID and 80 mg QD, respectively).

Among the 40 patients in the main cohort who received asciminib at the escalated dose of 200 mg QD, 17.5% had achieved MMR at Week 96.

In the exploratory cohort of patients already in MMR at baseline, the overall MMR maintenance rate at Week 48 was 93.3% (28/30 patients); 100% in the 40 mg BID arm and 87.5% in the 80 mg QD arm (Table 4). Two patients in the 80 mg QD arm discontinued treatment due to AEs before Week 48 and were counted as non-responders. At Week 96, MMR was maintained by 86.7% of patients (92.9% on 40 mg BID and 81.3% on 80 mg QD). The proportion of patients in MR^4^ rose from eight at baseline (3/14 on 40 mg BID and 5/16 on 80 mg QD) to 15 at Week 48 (7/14 on 40 mg BID and 8/16 on 80 mg QD) and 16 at Week 96 (7/14 on 40 mg BID and 9/16 on 80 mg QD); increases were also observed in MR^4.5^ rates (Table 4 and Supplementary Tables 12 and 13). Median duration of MR^4^ was not estimable.

**Table 4:** Response rates over time (exploratory cohort).

	MMR			MR^4^			MR^4.5^		
*n* (%)	Asciminib 40 mg BID *n* = 14	Asciminib 80 mg QD *n* = 16	All patients *N* = 30	Asciminib 40 mg BID *n* = 14	Asciminib 80 mg QD *n* = 16	All patients *N* = 30	Asciminib 40 mg BID *n* = 14	Asciminib 80 mg QD *n* = 16	All patients *N* = 30
At Baseline	14 (100)	16 (100)	30 (100)	3 (21.4)	5 (31.3)	8 (26.7)	2 (14.3)	2 (12.5)	4 (13.3)
At Week 48	14 (100)	14 (87.5)	28 (93.3)	7 (50.0)	8 (50.0)	15 (50.0)	5 (35.7)	3 (18.8)	8 (26.7)
At Week 96	13 (92.9)	13 (81.3)	26 (86.7)	7 (50.0)	9 (56.3)	16 (53.3)	5 (35.7)	4 (25.0)	9 (30.0)

*BID* twice daily, *CI* confidence interval, *MMR* major molecular response, *MR* molecular response, *QD* once daily, *RT-qPCR* reverse transcription quantitative polymerase chain reaction.

For MMR, patients without RT-qPCR assessment at 48 weeks were considered as non-responders at Week 48, unless both 36- and 60-week RT-qPCR assessments indicated that the patient was in MMR. For other response types, patients without RT-qPCR assessment at a time point were considered as non-responders at that time point.

### Patient-reported outcomes

Completion rates for the MDASI-CML full questionnaire were high among patients in the main cohort, ranging from 90.9% to 94.7% at different time points. MDASI-CML Symptom and Interference Total scores decreased slightly from baseline (with decreases generally ≤0.5) and quickly at Week 4, denoting a small improvement in patients’ symptoms and slightly reduced interference with their daily life activities; no worsening in scores from baseline was noted at any timepoint up to Week 96 (Supplementary Fig. 4). Similar completion rates (88.9% to 100%) and results were observed for patients already in MMR at baseline, although the reduction in scores appeared larger for Interference Total scores (Supplementary Fig. 5).

### Safety

In the main cohort, median exposure to asciminib was 115.1 weeks (range 1.4‒154.6) for all patients, 114.9 weeks (range 2.6‒151.6) for patients on 40 mg BID and 115.2 weeks (range 1.4‒154.6) for patients on 80 mg QD. Most patients (76.8%) were exposed to asciminib for at least 96 weeks (75.0% with asciminib 40 mg BID and 78.6% with asciminib 80 mg QD).

Overall, 94.0% of these patients experienced any-grade AEs (94.0% on both 40 mg BID and 80 mg QD) and 37.5% of patients experienced Grade ≥3 AEs (32.1% on 40 mg BID and 42.9% on 80 mg QD) (Supplementary Table 14); the most frequent AEs are presented in Table 5. AEs led to treatment discontinuation in 12 (7.1%) patients (seven patients in the 40 mg BID arm and five patients on the 80 mg QD arm); these AEs were thrombocytopenia (*n* = 3), decreased platelet count (*n* = 2), neutropenia, splenomegaly, pericardial effusion, blurred vision, electrocardiogram QT prolonged, hyperlipasemia, cerebrovascular accident, headache, and cough (reported in one patient each). AEs leading to dose adjustment or interruption were reported in 57 (33.9%) patients (29 patients on 40 mg BID and 28 patients on 80 mg QD).

**Table 5:** Most frequent AEs (reported in ≥5% of all patients, main cohort).

Preferred term, *n* (%)	Asciminib 40 mg BID *n* = 84		Asciminib 80 mg QD *n* = 84		All patients *N* = 168	
	All grades	Grade ≥ 3	All grades	Grade ≥ 3	All grades	Grade ≥ 3
Number of patients with at least one event	79 (94.0)	27 (32.1)	79 (94.0)	36 (42.9)	158 (94.0)	63 (37.5)
Thrombocytopenia^a^	11 (13.1)	7 (8.3)	17 (20.2)	10 (11.9)	28 (16.7)	17 (10.1)
Arthralgia	13 (15.5)	2 (2.4)	14 (16.7)	0	27 (16.1)	2 (1.2)
COVID-19	12 (14.3)	0	10 (11.9)	0	22 (13.1)	0
Fatigue	5 (6.0)	0	17 (20.2)	1 (1.2)	22 (13.1)	1 (0.6)
Headache	10 (11.9)	0	9 (10.7)	0	19 (11.3)	0
Pruritus	13 (15.5)	0	6 (7.1)	0	19 (11.3)	0
Lipase increased	9 (10.7)	1 (1.2)	8 (9.5)	1 (1.2)	17 (10.1)	2 (1.2)
Nausea	7 (8.3)	0	10 (11.9)	0	17 (10.1)	0
Diarrhea	8 (9.5)	0	8 (9.5)	0	16 (9.5)	0
Hypertension	9 (10.7)	1 (1.2)	7 (8.3)	2 (2.4)	16 (9.5)	3 (1.8)
Back pain	7 (8.3)	0	8 (9.5)	0	15 (8.9)	0
Neutropenia^b^	8 (9.5)	7 (8.3)	7 (8.3)	5 (6.0)	15 (8.9)	12 (7.1)
Upper respiratory tract infection	8 (9.5)	1 (1.2)	7 (8.3)	1 (1.2)	15 (8.9)	2 (1.2)
Abdominal pain	7 (8.3)	0	5 (6.0)	1 (1.2)	12 (7.1)	1 (0.6)
Alanine aminotransferase increased	9 (10.7)	2 (2.4)	3 (3.6)	1 (1.2)	12 (7.1)	3 (1.8)
Abdominal pain upper	5 (6.0)	0	6 (7.1)	0	11 (6.5)	0
Dry skin	3 (3.6)	0	8 (9.5)	1 (1.2)	11 (6.5)	1 (0.6)
Rash	5 (6.0)	0	6 (7.1)	0	11 (6.5)	0
Weight increased	4 (4.8)	1 (1.2)	7 (8.3)	0	11 (6.5)	1 (0.6)
Aspartate aminotransferase increased	8 (9.5)	1 (1.2)	2 (2.4)	0	10 (6.0)	1 (0.6)
Cough	4 (4.8)	0	5 (6.0)	0	9 (5.4)	0
Dyspnea	5 (6.0)	1 (1.2)	4 (4.8)	0	9 (5.4)	1 (0.6)
Myalgia	5 (6.0)	0	4 (4.8)	0	9 (5.4)	0
Pain in extremity	5 (6.0)	0	4 (4.8)	0	9 (5.4)	0

*AE* adverse event, *BID* twice daily, *CTCAE* Common Terminology Criteria for Adverse Events, *MedDRA* Medical Dictionary for Regulatory Activities, *QD* once daily.

Numbers (n) represent counts of patients.

A patient with multiple severity grades for an AE was only counted under the maximum grade. AEs occurring during treatment or within 30 days of last study medication are summarized. MedDRA version 27.1, CTCAE version 5.0.

^a^Includes thrombocytopenia and platelet count decreased.

^b^Includes neutropenia and neutrophil count decreased.

Among the 168 treated patients in the main cohort, pancreatic enzyme elevations were observed in 22 patients (13.1%) overall, 16.7% on 40 mg BID and 9.5% on 80 mg QD. Of these, there were two Grade 3 increased lipase (1.2%) and one Grade 3 hyperlipasemia (0.6%) events. Pancreatitis was not reported in this cohort. Eleven arterial occlusive events (AOEs) were reported (four on 40 mg BID and seven on 80 mg QD), of which four were Grade ≥3 (two in each arm).

One on-treatment death was reported in the 80 mg QD arm, caused by cerebrovascular accident on Day 11 in a 66-year-old female patient with concurrent type 2 diabetes mellitus, hypertension and a history of stroke two years before entering the study, who had been treated with losartan, bisoprolol, metformin and acetylsalicylic acid (as preventive therapy for stroke). The patient had previously received imatinib, nilotinib and dasatinib. This death was assessed by investigators as not suspected to be related to the study drug.

Among patients in the main cohort who received asciminib at 200 mg QD, 67.5% experienced at least one AE after dose escalation, with 10.0% reporting Grade ≥3 AEs. After dose escalation, the most common AE of special interest (AESI) was gastrointestinal toxicity (7/40 patients, 17.5%), with no Grade ≥3 events recorded. No AEs leading to discontinuation or deaths were reported for these patients.

Among patients in the exploratory cohort (already in MMR at baseline), median exposure to asciminib was 127.1 weeks (range 1.1‒151.1) for all patients, 127.1 weeks (range 77.7‒151.1) on 40 mg BID and 125.1 weeks (range 1.1‒146.0) on 80 mg QD; 90.0% of patients were exposed to asciminib for at least 96 weeks. Nearly all patients (96.7%) experienced any-grade AEs and 14 patients (46.7%, six on 40 mg BID and eight on 80 mg QD) experienced Grade ≥3 AEs (Table 6 and Supplementary Table 15). Two patients had AEs leading to discontinuation (Grade 3 acute pancreatitis and Grade 3 lipase increase, *n* = 1 each, both in the 80 mg QD arm) and nine patients had AEs leading to dose adjustment or interruption (five patients on 40 mg BID and four patients on 80 mg QD). Lipase increase was reported in four patients (13.3%) and amylase increase was reported in two patients (6.7%); of these, two increased lipase events were Grade ≥3 (6.7%).

**Table 6:** Most frequent AEs (reported in ≥10% of all patients, exploratory cohort).

Preferred term, *n* (%)	Asciminib 40 mg BID *n* = 14		Asciminib 80 mg QD *n* = 16		All patients *N* = 30	
	All grades	Grade ≥ 3	All grades	Grade ≥ 3	All grades	Grade ≥ 3
Number of patients with at least one event	13 (92.9)	6 (42.9)	16 (100)	8 (50.0)	29 (96.7)	14 (46.7)
Arthralgia	4 (28.6)	0	4 (25.0)	0	8 (26.7)	0
Pruritus	3 (21.4)	0	5 (31.3)	0	8 (26.7)	0
Headache	2 (14.3)	0	5 (31.3)	0	7 (23.3)	0
COVID-19	3 (21.4)	0	3 (18.8)	0	6 (20.0)	0
Diarrhea	1 (7.1)	0	5 (31.3)	0	6 (20.0)	0
Myalgia	2 (14.3)	0	4 (25.0)	1 (6.3)	6 (20.0)	1 (3.3)
Aspartate aminotransferase increased	2 (14.3)	0	3 (18.8)	0	5 (16.7)	0
Abdominal pain	2 (14.3)	0	2 (12.5)	0	4 (13.3)	0
Alanine aminotransferase increased	1 (7.1)	0	3 (18.8)	0	4 (13.3)	0
Dyspnea	2 (14.3)	0	2 (12.5)	0	4 (13.3)	0
Lipase increased	2 (14.3)	1 (7.1)	2 (12.5)	1 (6.3)	4 (13.3)	2 (6.7)
Creatine phosphokinase increased	2 (14.3)	1 (7.1)	1 (6.3)	0	3 (10.0)	1 (3.3)
Bronchitis	0	0	3 (18.8)	0	3 (10.0)	0
Arterial hypertension	1 (7.1)	0	2 (12.5)	2 (12.5)	3 (10.0)	2 (6.7)
Low-density lipoprotein increased	1 (7.1)	0	2 (12.5)	0	3 (10.0)	0
Nausea	0	0	3 (18.8)	0	3 (10.0)	0
Pyrexia	0	0	3 (18.8)	0	3 (10.0)	0
Respiratory tract infection	1 (7.1)	0	2 (12.5)	0	3 (10.0)	0
Upper respiratory tract infection	0	0	3 (18.8)	0	3 (10.0)	0
Urinary tract infection	3 (21.4)	0	0	0	3 (10.0)	0

Numbers (n) represent counts of patients. A patient with multiple severity grades for an AE was only counted under the maximum grade. AEs occurring during treatment or within 30 days of the last study medication are summarized. MedDRA version 27.1, CTCAE version 5.0.

*AE* adverse event, *BID* twice daily, *CTCAE* Common Terminology Criteria for Adverse Events, *MedDRA* Medical Dictionary for Regulatory Activities, *QD* twice daily.

One death was reported in this cohort in the 40 mg BID arm, occurring on Day 552 of the study (52 days after the last dose of asciminib had been received). The patient was a 66-year-old male, and the cause of death was hypoxemic respiratory failure. This death was also assessed by investigators as not suspected to be related to the study drug.

### BCR::ABL1 mutations

The presence of *BCR::ABL1* mutations was assessed at baseline in patients from the main cohort (Supplementary Table 16). Overall, 25 patients (14.8%) were identified as harboring a mutation before treatment with asciminib started. The most common mutation was Y253H (*n* = 6), followed by M244V and T315I (*n* = 4 each), and E255K and G250E (*n* = 3 each). Among patients with *BCR::ABL1* mutations at baseline, 15/25 patients discontinued treatment (M244V and T315I, *n* = 4 each; E255K, *n* = 3); none of these patients had achieved MMR. Of the ten patients with *BCR::ABL1* mutations at baseline still receiving treatment at data cutoff (Y253H, *n* = 6), seven had achieved MMR by Week 48 and one by Week 96, while the remaining two patients had *BCR::ABL1*^IS^ < 1% at Week 84. Patients harboring Y253H (*n* = 2), F317L (*n* = 1), E255K (*n* = 1), and M244V/G250E (*n* = 1) mutations had their asciminib dose escalated to 200 mg QD (Supplementary Table 16).

Nine patients had newly emerging mutations detected post-baseline (M244V, *n* = 3; T315I, P465A, A68P/F317L, K294E, A433T, F359V, *n* = 1 each) (Supplementary Table 17). Of these, seven patients discontinued treatment, and two received asciminib at the increased dose of 200 mg QD.

All but one of the seven patients with an M244V mutation at baseline or emerging on treatment discontinued asciminib. On the other hand, all six patients harboring the Y253H mutation at baseline were receiving treatment with asciminib at data cutoff; four of these patients achieved MMR at Week 48, and the remaining two had *BCR::ABL1*^IS^ < 1%.

## Discussion

The results of the ASC4OPT study demonstrate that asciminib at both 40 mg BID and 80 mg QD doses is highly efficacious and shows favorable safety and tolerability in patients with CML-CP previously treated with two or more TKIs, including those with suboptimal response on previous TKIs. Asciminib also showed good efficacy and favorable safety in patients already in MMR at baseline. These findings complement those of the ASCEMBL study in a 3 L+ patient population [8].

Most patients had discontinued their previous TKI due to treatment resistance. The MMR rates observed at Week 48 and Week 96—numerically higher than those observed in the ASCEMBL study (29.3% at Week 48 and 37.6% at Week 96 for asciminib 40 mg BID) [9, 24]—support the efficacy of asciminib in patients with suboptimal response to ATP-competitive TKIs and raise the possibility that switching treatment for patients in suboptimal response may increase the number of patients who respond. Response rates in ASC4OPT at Week 48 were also higher than those reported for bosutinib (MMR, 13.2% at Week 48 in ASCEMBL, and 15% after a median of 8.3 months of treatment) [24, 25] and ponatinib (MMR, 34% with 45 mg QD in the PACE trial; *BCR::ABL1*^IS^ ≤ 1%, 29.0% with 30 mg QD, and 23.1% with 15 mg QD in the OPTIC trial) [26, 27] in a similar population of heavily pretreated patients. MMR rates increased from Week 48 to Week 96, showing the benefits of staying on treatment in the longer term.

One of the aims of the ASC4OPT study was to assess the efficacy and safety of asciminib in an expanded patient population, including not only those patients in failure but also those with warning response as per ELN 2020 definitions [11]. The ASC4OPT patient population thus included patients with less advanced disease and lower risk of progression than those enrolled in ASCEMBL [8]. This may explain the higher MMR rates reported in ASC4OPT, as an earlier switch to asciminib may be associated with improved response. A relevant proportion of these pretreated patients achieved deep molecular responses at Week 48 and Week 96 with both asciminib dosing regimens; these proportions were also numerically higher than the MR^4^ and MR^4.5^ rates achieved with asciminib in ASCEMBL [9, 28]. These results suggest that these patients have increased chances of attempting treatment-free remission (TFR).

Dose escalation to 200 mg QD appeared to be a suitable option for patients in the main cohort who did not achieve MMR at Week 48 or lost it between Week 48 and Week 108. Although the MMR rate for this subpopulation was modest (17.5% at Week 96), this likely reflects the challenges in the management of patients who have received multiple lines of treatment.

Patients treated with ≥2 TKIs and intolerant of their previous TKI treatment represent a clinical challenge, as the number of treatment options available to them is limited. Almost all ASC4OPT patients in this group (who were already in MMR at baseline) who were still on asciminib at Week 48 maintained this level of response, and the proportion of patients in MR^4^ increased over time through Week 96. Although most of these patients experienced AEs on asciminib (including a higher proportion of patients experiencing Grade ≥3 AEs compared with the main cohort ASC4OPT patients), few discontinuations due to AEs were observed. These results underscore the importance of remaining on treatment in the longer term and suggest good tolerability with asciminib treatment that was not achieved with ATP-competitive TKIs, potentially leading to further benefits in terms of QoL and TFR eligibility.

Although MMR is an established treatment goal for most CML clinical trials, achieving MMR is not always feasible in later lines of therapy [29]. Achievement of *BCR::ABL1*^IS^ ≤ 1% at 12 months has been associated with improved long-term outcomes, including a reduced risk of progression to the accelerated phase/blast phase and longer overall survival [30]. In ASC4OPT, nearly two-thirds of patients treated with both 40 mg BID and 80 mg QD achieved *BCR::ABL1*^IS^ ≤ 1% as early as Week 24, a higher proportion than that reported in ASCEMBL (49.0%) [8] and those reported with ponatinib as third-line treatment in the PACE and OPTIC trials at similar time points [26, 31].

Although the ASC4OPT study was not powered to assess differences between dosing regimens, the results support clinical data showing the similar efficacy of the 40 mg BID and 80 mg QD asciminib dosing regimens in the asciminib phase 1 study [19]. In addition, the 80 mg QD dosing schedule has been used in the ASC4FIRST trial, demonstrating superior efficacy and favorable safety versus all standard-of-care frontline TKIs [22].

PROs showed a slight but rapid improvement in scores related to symptom severity and interference with daily activities at Week 4 after asciminib start, maintained up to Week 96. This suggests that QoL was sustained with asciminib treatment, although longer-term results are needed to assess whether asciminib can improve QoL in a clinically meaningful way.

The safety profile of asciminib at 80 mg QD was similar to that of the 40 mg BID dosing schedule, with no new or unexpected safety signals observed. Treatment discontinuation due to AEs was infrequent and occurred early with asciminib, similar to what was reported in the ASCEMBL trial [8, 9], which suggests that most patients who remain on treatment after 6 months of starting asciminib will likely stay on therapy in the longer term. Pancreatic toxicity events were infrequent and mostly related to asymptomatic enzyme elevations, leading to discontinuation in only three patients; two of these patients were already in MMR at baseline and had discontinued their previous TKI due to intolerance. The frequency of AOEs was similar to that reported in ASCEMBL (6.5% vs 5.1%, respectively) [9]; given that both trials enrolled patient populations with comparable exposure to ATP-competitive TKIs, a key driver of cardiovascular risk, the observed rates of AOEs likely reflect the comparable baseline susceptibility conferred by prior multi‑line TKI therapy. Patients receiving TKI therapy, particularly those with existing cardiovascular risk factors, should undergo baseline risk assessment and be closely monitored. Importantly, safety at the higher dose of 200 mg QD remained favorable, with no new safety signals or discontinuations due to AEs.

Overall, 25 patients had at least one *BCR::ABL1* mutation at baseline, and ten emergent mutations were detected post-baseline in nine patients. The majority of mutations identified at baseline did not involve the myristoyl pocket or cause allosteric shifts to more active BCR::ABL1 conformations despite asciminib binding (such as M244V). Correlation with response suggests that M244V confers resistance to asciminib, given that nearly all patients harboring this mutation discontinued treatment. The presence of the Y253H mutation did not appear to affect asciminib efficacy to the same extent; all patients with this mutation remained in the study at data cutoff, although two patients required dose escalation. Of note, five out of the six patients with a Y253H mutation at baseline had received nilotinib as their most recent TKI.

The main limitation of this study is that it was not powered to compare the two asciminib dosing regimens, which means that small, significant differences in efficacy and safety between the two dosing schedules may not have been detected. Although the number of patients already in MMR at baseline was likely too low to provide generalizable conclusions for this patient population, the data provide useful insights into this group of patients with an unmet need.

Overall, the results complement those of the ASCEMBL study to support asciminib as a standard of care in non-optimally responding patients with CML and in those patients who discontinued previous treatments due to intolerance. Long-term results from ASC4OPT will provide further insights into the optimization of asciminib treatment.

## Supplementary information

Supplemental material

## Data Availability

Novartis is committed to sharing access to patient-level data and supporting clinical documents from eligible studies with qualified external researchers. These requests are reviewed and approved by an independent review panel based on scientific merit. All data provided are anonymized to respect the privacy of patients who have participated in the trial in line with applicable laws and regulations. This trial data availability is according to the criteria and process described on www.clinicalstudydatarequest.com.
