# Supplementary material for: ASC4OPT: asciminib treatment optimization study in patients with chronic myeloid leukemia in chronic phase previously treated with two or more tyrosine kinase inhibitors
Source: Leukemia. 2026 Apr 23;40(6):1296–305. doi: 10.1038/s41375-026-02965-8 (PMC13233290; doi:10.1038/s41375-026-02965-8)
Supplement: Supplementary file 1 — Supplemental material [file 41375_2026_2965_MOESM1_ESM.pdf]

Supplementary Materials

ASC4OPT: Asciminib treatment optimization study in patients with chronic myeloid leukemia in chronic phase previously treated with two or more tyrosine kinase inhibitors

Andreas Hochhaus[1], Philipp le Coutre[2], Dragana Milojkovic[3], Dennis Dong Hwan Kim[4], Soo Min Lim[5], Carolina Pavlovsky[6], Thanh Nguyen[7], Franck Emmanuel Nicolini[8], Beatriz Moiraghi[9], Sebastian Grosicki[10], Chi Dung Phu[11], Gabriel Etienne[12], Fernando Marco de Lucas[13], Rosa Maria Ayala Diaz[14], Massimo Breccia[15], Charles Chuah[16], Roberto Abi Rached[17], Himanshu Pokhriyal[18], Aswin IC[18], Peter Schuld[17], Virginia Pilipovic[17], Franz Alisch[17], Carla Maria Boquimpani[19]

[1]Klinik für Innere Medizin II, Hematology/Oncology, Universitätsklinikum Jena and Comprehensive Cancer Center Central Germany, Campus Jena, Jena, Germany; [2]Department of Oncology and Hematology, Charité-Universitätsmedizin Berlin, Berlin, Germany; [3]Centre for Haematology, Department of Immunology and Inflammation, Imperial College London, and Department of Clinical Haematology, Imperial College Healthcare NHS Trust, London, UK; [4]Princess Margaret Cancer Centre, University Health Network, University of Toronto, Toronto, Canada; [5]Sultanah Aminah Hospital, Johor Bahru, Malaysia; [6]FUNDALEU, Buenos Aires, Argentina; [7]National Institute of Hematology and Blood Transfusion, Vietnam; [8]Department of Hematology & INSERM U1052, Centre Léon Bérard and CRCL, Lyon, France; [9]Hospital Jose Maria Ramos Mejia, Buenos Aires, Argentina; [10]Department of Hematology and Cancer Prevention, Faculty of Health Sciences in Bytom, Medical University of Silesia, Katowice, Poland; [11]Blood Transfusion Hematology Hospital, Ho Chi Minh City, Vietnam; [12]Hematology Department, Institut Bergonié, Bordeaux, France; [13]Servicio de Hematología, Hospital Universitario Basurto, Osakidetza, Bilbao, Spain; [14]Haematological Malignancies Clinical Research Unit, Hospital 12 de Octubre Universidad Complutense, CNIO, CIBERONC, and Spanish National Cancer Research Center (CNIO), Madrid, Spain; [15]Hematology, Department of Translational and Precision Medicine, Sapienza University of Rome, Rome, Italy; [16]Singapore General Hospital, Duke-NUS Medical School, Singapore, Singapore; [17]Novartis Pharma AG, Basel, Switzerland; [18]Novartis Healthcare Pvt. Ltd., Salarpuria-Sattva Knowledge City, Hyderabad, India; [19]HEMORIO, State Institute of Hematology Arthur de Siquiera Cavalcanti, Rio de Janeiro, Brazil

Contents

Supplementary Methods 3

Inclusion and exclusion criteria 3

Sample size 4

Analysis sets 4

Definitions 5

Dose escalation 7

Dose modifications 7

Criteria for dose reduction / interruption / discontinuation and re-initiation of treatment for adverse drug reactions 8

Propensity Scores Weighting analysis 16

References 17

Supplementary Figures 18

Supplementary Figure 1. Study design 18

Supplementary Figure 2. Sequence of prior TKIs in the main cohort. 19

Supplementary Figure 3. Kaplan-Meier estimate of duration of MMR among patients who achieved MMR in the main cohort. 20

Supplementary Figure 4. MDASI-CML Symptom Total Score Derived (A) and Interference Total Score Derived (B) scores (main cohort). 21

Supplementary Figure 5. MDASI-CML Symptom Total Score Derived (A) and Interference Total Score Derived (B) scores (exploratory cohort). 23

Supplementary Tables 25

Supplementary Table 1. Patient demographics and disease characteristics for the patients who received asciminib 200 mg QD in the main cohort. 25

Supplementary Table 2. MMR rate at scheduled times in the main cohort excluding patients with the T315I mutation detected after treatment start. 26

Supplementary Table 3. MMR rate by scheduled times in the main cohort excluding patients with the T315I mutation detected after treatment start 27

Supplementary Table 4. Comparison of baseline prognostic factors for ad-hoc MMR rate analysis at Week 48 in the main cohort excluding patients with the T315I mutation at baseline. 28

Supplementary Table 5. Analysis of MMR rates on asciminib 40 mg BID vs 80 mg QD in ASC4OPT vs 40 mg BID in ASCEMBL using propensity score weighting (main cohort excluding patients with the T315I mutation at baseline). 29

Supplementary Table 6. MR^4 rate at scheduled times (main cohort excluding patients with the T315I mutation at baseline). 30

Supplementary Table 7. MR^4 by scheduled times (main cohort excluding patients with the T315I mutation at baseline) 31

Supplementary Table 8. $MR^{4.5}$ at scheduled times (main cohort excluding patients with the T351I mutation at baseline) 32

Supplementary Table 9. $MR^{4.5}$ by scheduled times (main cohort excluding patients with the T351I mutation at baseline) 33

Supplementary Table 10. *BCR::ABL1*^{IS} ≤1% rate at scheduled times (main cohort excluding patients with the T315I mutation at baseline). 34

Supplementary Table 11. *BCR::ABL1*^{IS} ≤1% rate by scheduled times (main cohort excluding patients with the T315I mutation at baseline). 35

Supplementary Table 12. MR^4 at scheduled times (exploratory cohort)..36
Supplementary Table 13. MR$^{4.5}$ at scheduled times (exploratory cohort).....................................37
Supplementary Table 14. Overview of adverse events (main cohort). 38
Supplementary Table 16. Mutations detected at baseline by NGS (main cohort). 40
Supplementary Table 17. Post-baseline mutations detected by NGS (main cohort).....................41

Supplementary Methods

Inclusion and exclusion criteria

Enrollment took place across 48 sites in Argentina, Austria, Brazil, Canada, France, Germany, Greece, Italy, Malaysia, Oman, Poland, Singapore, South Korea, Spain, United Kingdom and Vietnam.

Participants eligible for inclusion in this study must have met all of the following criteria:

- Signed informed consent obtained prior to participation in the study
- Male or female patients with a diagnosis of chronic myeloid leukemia in chronic phase (CML-CP) ≥18 years of age
    - Met all of the following laboratory values at the screening visit (<15% blasts in peripheral blood and bone marrow; <30% blasts plus promyelocytes in peripheral blood and bone marrow; <20% basophils in the peripheral blood; ≥50 x 10^9/L [≥ 50,000/mm^3] platelets)
- No evidence of extramedullary leukemic involvement, with the exception of hepatosplenomegaly
- Prior treatment with a minimum of 2 prior TKIs (i.e. imatinib, nilotinib, dasatinib, bosutinib, radotinib or ponatinib)
- Eastern Cooperative Oncology Group (ECOG) performance status (PS) of 0, 1, or 2
- Evidence of typical *BCR::ABL1* transcripts [e14a2 and/or e13a2] at the time of screening. For patients in the exploratory cohort, historical laboratory results could be used
- Patients were considered as being in warning or failure response (adapted from the 2020 ELN recommendations) or intolerant to the most recent tyrosine kinase inhibitor (TKI) therapy at the time of screening.

Warning response was defined as *BCR::ABL1* >10% on the International Scale (IS) three months after treatment initiation; *BCR::ABL1*^{IS} >1–10% six months after treatment initiation; *BCR::ABL1*^{IS} >0.1–1%

12 months after treatment initiation; *BCR::ABL1*^{IS} >0.1–1%, loss of major molecular response (MMR, >0.1% with 5-fold increase of *BCR::ABL1* transcripts) At any time after treatment initiation.

Failure response was defined as *BCR::ABL1*^{IS} >10% three months after treatment initiation if confirmed within 1–3 months; *BCR::ABL1*^{IS} >10% six months after treatment initiation; *BCR::ABL1*^{IS} >1% 12 months after treatment initiation; *BCR::ABL1*^{IS} >1%, emergence of resistance mutations, high-risk additional chromosomal abnormalities (ACAs)[2] at any time after treatment initiation.

Intolerance was defined as:

- Non-hematologic intolerance: Patients with grade 3 or 4 toxicity while on therapy, or with persistent grade 2 toxicity, unresponsive to optimal management, including dose adjustments (unless dose reduction was not considered in the best interest of the patient if response was already suboptimal)
- Hematologic intolerance: Patients with grade 3 or 4 toxicity (absolute neutrophil count [ANC] or platelets) while on therapy that is recurrent after dose reduction to the lowest doses recommended by manufacturer

Sample size

A sample size of 156 patients was estimated to have 80% power to reject the null hypothesis proportion of 23% (which was the upper limit of the 95% exact binomial confidence interval of the MMR rate at Week 48 observed in the bosutinib arm in the ASCEMBL study)[3] at 0.025 one-sided level of significance if the true rate (under the alternative hypothesis) was 33% (considering a clinically meaningful difference ≥10%) using an exact test for single proportion.

Analysis sets

The Full Analysis Set (FAS) comprised all participants to whom study treatment was assigned and who received at least one dose of study treatment except the additional patients who were intolerant to the last TKI and were in MMR at baseline.

The Full Analysis Set 2 (FAS 2) comprised only the patients in the exploratory cohort who were intolerant to the last TKI and in MMR at baseline and to whom study treatment was assigned and received at least one dose of study treatment.

The Safety Set (SAF) included all participants who received at least one dose of study treatment except the additional patients who were intolerant to the last TKI and were in MMR at baseline.

The Safety Set 2 (SAF 2) included only the patients in the exploratory cohort who were intolerant to the last TKI and in MMR at baseline and who received at least one dose of study treatment.

**Analysis set**	**Asciminib 40 mg BID n=99 n (%)**	**Asciminib 80 mg QD n=100 n (%)**	**All patients N=199 n (%)**
Full analysis set	85 (85.9)	84 (84.0)	169 (84.9)
Full analysis set 2	14 (14.1)	16 (16.0)	30 (15.1)
Safety set	84 (84.8)	84 (84.0)	168 (84.4)
Safety set 2	14 (14.1)	16 (16.0)	30 (15.1)

Definitions

Time to MMR was defined as the time from the date of enrolment to the date of the first documented MMR (date of first documented MMR - date of randomization +1)

Duration of MMR was defined as the time from the date of first documented MMR to the earliest date of loss of MMR, progression to accelerated phase (AP) or blast phase (BP), or CML-related death

Loss of MMR was defined as increase of *BCR::ABL1*/*ABL* to > 0.1% by IS in association with a ≥5-fold rise in *BCR::ABL1* transcripts from the lowest value achieved on study treatment and replicated by a second analysis of the same sample. Loss of MMR was confirmed by subsequent sample analysis within 4 to 6 weeks showing loss of MMR associated with a ≥5-fold rise in *BCR::ABL1* from the lowest value achieved on study treatment, unless it was associated with confirmed loss of complete hematologic response (CHR) or loss of complete cytogenetic response (CCyR) or progression to AP/BP or CML-related death.

Cytogenetic response was defined as the following (a review of a minimum of 20 metaphases was required):

- Major (MCyR): 0 to 35% Ph+ metaphases
- Complete (CCyR): 0% Ph+ metaphases
- Partial (PCyR): >0 to 35% Ph+ metaphases
- Minor (mCyR): >35 to 65% Ph+ metaphases
- Minimal: >65 to 95% Ph+ metaphases
- None: >95 to 100% Ph+ metaphases.

Bone marrow aspirate for cytogenetic analyses was performed at screening/baseline (performed up to 56 days prior to Week 1 Day 1), at Week 48 and at end of treatment (only required if the patient discontinues from the study early due to lack/loss of response). If bone marrow assessments had been performed before the main informed consent was signed but within 56 days of Week 1 Day 1, no further bone marrow sampling was required at screening and local assessment data were collected.

CHR was defined as all of the following present for ≥ 4 weeks: white blood cell (WBC) count <10 x 10^9/L; platelet count <450 x 10^9/L; basophils <5%; no blasts and promyelocytes in peripheral blood; myelocytes + metamyelocytes < 5% in peripheral blood; no evidence of extramedullary disease, including spleen and liver.

Disease progression was defined as follows:

- CML-related death (any death during treatment or follow-up if the principal cause of death is marked as “study indication” in the electronic case report form (eCRF) by the investigator, or if the death occurred subsequent to documented progression to AP/BC and the cause of death is reported as “unknown” or not reported by the investigator)
- AP as defined by any of the following:
    - ≥15% blasts in the peripheral blood or bone marrow aspirate, but < 30% blasts in both the peripheral blood and bone marrow aspirate
    - ≥30% blasts plus promyelocytes in peripheral blood or bone marrow aspirate
    - ≥20% basophils in the peripheral blood
    - Thrombocytopenia (<100 x 10^9/L) that is unrelated to therapy
- Blast crisis (BC) as defined by any of the following:

- ≥30% blasts in peripheral blood or bone marrow aspirate
- Appearance of extramedullary involvement other than hepatosplenomegaly proven by biopsy (i.e., chloroma).

Any value of AP or BC within the first 4 weeks of study treatment is not defined as progression to AP/BC within the study unless the patient discontinues study treatment due to progression.

Dose escalation

For patients not in MMR at 48 weeks or losing the response after the week 48 assessments up to week 108, a dose escalation to 200 mg QD was considered for patients on 40 mg BID or 80 mg QD, if in the investigator's opinion the patient may have benefited from the escalation. In addition, there must not be any grade 3 or 4 toxicity while on therapy, or persistent grade 2 toxicity, possibly related to asciminib and unresponsive to optimal management.

Dose modifications

For participants who do not tolerate the protocol-specified dosing schedule, dose interruptions, and/or reductions are either recommended or mandated in order to allow participants to continue the study treatment.

<table>
<tr><td colspan="3">Dose reduction schedule</td></tr>
<tr><td></td><td>Starting dose level 0</td><td>Dose level – 1</td></tr>
<tr><td rowspan="3">Asciminib</td><td>40 mg BID (total daily dose 80 mg)</td><td>20 mg BID (total daily dose 40 mg)</td></tr>
<tr><td>80 mg QD (total daily dose 80 mg)</td><td>40 mg QD (total daily dose 40 mg)</td></tr>
<tr><td>200 mg QD (total daily dose 200 mg)</td><td>80 mg QD or 40 mg BID (total daily dose 80 mg)**</td></tr>
<tr><td colspan="3">*Dose reduction should be based on the worst toxicity demonstrated at the last dose.
**Patients on 200 mg QD needing a dose de-escalation will revert to 80 mg total daily dose of asciminib, according to pre-escalation dosing schedule (QD or BID regimen assigned at enrolment).</td></tr>
<tr><td colspan="3">Asciminib dose reduction below total daily dose of 40 mg (20 mg BID or 40 mg QD) is not allowed. 20 mg tablets will be dispensed to patients having 20 mg BID regimen in the instance of dose reduction.</td></tr>
</table>

Criteria for dose reduction / interruption / discontinuation and re-initiation of treatment for adverse drug reactions

**Worst toxicity CTCAE Version 5.0**	**Asciminib**
Neutropenia (absolute neutrophil count, ANC)	
Grade 1 (ANC <lower limit of normal [LLN]–1.5 x 10^9/L)	**Recommendation**: Maintain dose level
Grade 2 (ANC <1.5–1.0 x 10^9/L)	**Recommendation**: Maintain dose level
Grade 3 (ANC <1.0–0.5 x 10^9/L)	**Mandatory**: Hold dose until resolved to grade ≤2 (recheck complete blood count [CBC] 2x/week), then:<br>if resolved in ≤14 days, then maintain dose level<br>If resolved in >14 days, then reduce dose 1 dose level
Grade 4 (ANC <0.5 x 10^9/L)	**Mandatory**: Hold dose until resolved to grade ≤2, (recheck CBC 2x/week), then:<br>if resolved in ≤14 days, then maintain dose level<br>if resolved in >14 days, then reduce dose 1 dose level
Febrile neutropenia (ANC <1.0 x 10^9/L, fever ≥38.5°C)	**Mandatory:** Hold dose until resolved, then reduce dose 1 dose level
Thrombocytopenia	
Grade 1 (platelet count [PLT] <LLN–75 X 10^9/L)	**Recommendation**: Maintain dose level
Grade 2 (PLT <75–50 x 10^9/L)	**Recommendation**: Maintain dose level
Grade 3 (PLT <50–25 x 10^9/L)	**Mandatory:** Hold dose until resolved to grade ≤2 (recheck CBC 2x/week), then:<br>if resolved in ≤14 days, then maintain dose level<br>if resolved in >14 days, then reduce dose 1 dose level
Grade 4 (PLT <25 x 10^9/L)	**Mandatory:** Hold dose until resolved to grade ≤2 (recheck CBC 2x/week), then:

	if resolved in ≤14 days, then maintain dose level<br>if resolved in >14 days, then reduce dose 1 dose level
Recurrence of any cytopenia	**Recommendation:** Hold dose until resolved to grade ≤2, then maintain current dose level<br>**For recurrent Grade 3/4 cytopenia, please refer to above guidelines for neutropenia and thrombocytopenia; no further dose reduction is allowed for already reduced dose.**
**Non-hematologic adverse reactions except where further specified in individual sections**	
Grade 1	**Recommendation**: Maintain dose level
Grade 2	**Recommendation:** Hold dose until resolved to grade ≤1, then maintain dose level
Grade 3	**Mandatory:** Hold dose until resolved to grade ≤1, then reduce dose 1 dose level
Grade 4	**Mandatory**: Permanently discontinue patient from treatment
**Investigations (renal)**	
Serum creatinine	
Grade 1 (>upper limit of normal [ULN]—1.5 x ULN)	**Recommendation:** Maintain dose level
Grade 2 (>1.5—3.0 x ULN)	**Recommendation:** Hold dose until resolved to grade ≤1 or baseline, then maintain dose level
Grade 3 (>3.0—6.0 x ULN)	**Mandatory:** Permanently discontinue patient from treatment
Grade 4 (>6.0 x ULN)	**Mandatory:** Permanently discontinue patient from treatment
**Investigations (hepatic)**	
Isolated total bilirubin elevation	
>ULN–1.5 x ULN, if baseline was normal	**Recommendation:** Maintain dose level
>1.5—3.0 x ULN if baseline was normal	**Recommendation:** Maintain dose. Repeat Liver Function Tests (LFT) within 48—72 hours then monitor LFTs[b] weekly, or more

	frequently if clinically indicated, until resolved to ≤1.5 x ULN or baseline:<br>if resolved in ≤14 days, then maintain dose level<br>if resolved in >14 days, then reduce dose 1 dose level
>3.0—10.0 x ULN (irrespective of the baseline levels) *	**Mandatory:** Hold dose. Repeat LFTs within 48—72 hours then monitor LFTs[b] weekly, or more frequently if clinically indicated, until resolved to ≤1.5 x ULN or baseline:<br>if resolved in ≤14 days, then reduce dose 1 dose level<br>if resolved in >14 days, then discontinue patient from treatment.<br>The patient should be monitored weekly (including LFTs[b]), or more frequently if clinically indicated, until total bilirubin level has resolved to baseline or has stabilized over 4 weeks
>10.0 x ULN (irrespective of the baseline levels) *	See footnote**** - otherwise discontinue study treatment<br>The patient should be monitored weekly (including LFTs[b]), or more frequently if clinically indicated, until total bilirubin level has resolved to baseline or has stabilized over 4 weeks
Isolated aspartate aminotransferase (AST) or alanine aminotransferase (ALT) elevation	
If normal at baseline:	
>ULN—3.0 x ULN	**Recommendation:** Maintain dose level<br>Repeat liver tests within 48—72 hours, then monitor weekly until recovery to ≤Grade 1 or to baseline
>3.0—5.0 x ULN	**Recommendation:** Maintain dose level. Repeat LFTs[b] as soon as possible, preferably within 48—72 hours from awareness of the abnormal results; if abnormal lab values are confirmed upon the repeat test, then monitor LFTs[b] weekly, or more frequently if clinically indicated, until resolved to ≤3.0 x ULN
>5.0—10.0 x ULN	**Mandatory**: Omit dose. Repeat LFTs[b] as soon as possible, preferably within 48—72 hours from awareness of the abnormal results; monitor LFTs[b] weekly, or more frequently if clinically indicated, until resolved to ≤3.0 x ULN. Then:

	If resolved in ≤14 days, resume at prior dose level<br>If resolved in >14 days, resume with reduced dose 1 dose level
>10.0–20.0 x ULN	**Mandatory: Omit dose**. Repeat LFTs[b] as soon as possible, preferably within 48–72 hours from awareness of the abnormal results; monitor LFTs[b] weekly, or more frequently if clinically indicated, until resolved to ≤ baseline. Then resume with reduced dose 1 dose level
>20.0 x ULN	**Mandatory:** Permanently discontinue
If elevated at baseline:	
>Baseline - 3.0 x Baseline AND ≤5 x ULN	Recommendation: Maintain dose level
>3.0 x Baseline AND >5.0 x ULN (duration less than 2 weeks)	Recommendation: Maintain dose level. Repeat LFTs[b] as soon as possible, preferably within 48–72 hours from awareness of the abnormal results; if abnormal lab values are confirmed upon the repeat test, then monitor LFTs[b] weekly, or more frequently if clinically indicated, until resolved to ≤ ULN or baseline
>3.0 x Baseline AND >5.0 x ULN (duration more than 2 weeks):	Mandatory: Omit dose. Repeat LFTs[b] as soon as possible, preferably within 48–72 hours from awareness of the abnormal results; if abnormal lab values are confirmed upon the repeat test, then monitor LFTs[b] weekly, or more frequently if clinically indicated, until resolved to ≤ ULN or baseline. If resolved, resume with reduced 1 dose level.
>5.0 x Baseline AND >8.0 x ULN (irrespective of the duration):	Mandatory: Omit dose. Repeat LFTs[b] as soon as possible, preferably within 48–72 hours from awareness of the abnormal results; if abnormal lab values are confirmed upon the repeat test, then monitor LFTs[b] weekly, or more frequently if clinically indicated, until resolved to ≤ ULN or baseline. If resolved, resume with reduced 1 dose level.
>20.0 x ULN	Permanently discontinue

Combined[c] elevations of AST or ALT and total bilirubin	
For patients with normal baseline ALT and AST and total bilirubin value:<br><br>AST or ALT >3.0 x ULN combined with total bilirubin >2.0 x ULN without evidence of cholestasis[d]<br>For patients with elevated baseline AST or ALT or total bilirubin value<br>[AST or ALT >3 x baseline OR [AST or ALT >8.0 x ULN], whichever is lower combined with total bilirubin >2 x baseline AND >2.0 x ULN<br>Note: For participants with Gilbert's syndrome, at least 2-fold increase in direct bilirubin	**Mandatory:** Hold dose<br><br>Repeat liver tests as soon as possible, preferably within 48 hours from awareness of the abnormal results, then with weekly monitoring of LFTs[b], or more frequently if clinically indicated, until AST, ALT, or bilirubin have resolved to baseline or stabilization over 4 weeks. Please refer to Section 6.6.4.1 for additional follow-up evaluations as applicable.<br><br>If drug-induced liver injury (DILI) confirmed: permanently discontinue patient from study drug treatment<br><br>If not DILI – interrupt treatment. Treat identified cause according to institutional guidelines. If resolved, then reduce dose 1 dose level, if cause is treatment related.
**Investigation (metabolic)**	
Amylase and/or lipase elevation	
Grade 1: > ULN–1.5 x ULN	**Recommendation:** Maintain dose level, measure 2x per week
Grade 2: >1.5–2.0 x ULN;<br>>2.0–5.0 x ULN and<br>Asymptomatic	**Recommendation:** Maintain dose level, measure 2x per week
Grade 3: >2.0–5.0 x ULN with signs or symptoms; >5.0 x ULN and asymptomatic	**Mandatory:** Hold dose until resolved to Grade ≤1 or baseline, then: If resolved in ≤7 days, then reduce dose 1 dose level<br>If resolved in >7 days, then discontinue treatment and obtain appropriate imaging (i.e., MRI, CT scan, or ultrasound) **
Grade 4: > 5.0 x ULN and with signs or symptoms	**Mandatory:** Permanently discontinue patient from treatment. Obtain appropriate imaging (i.e., MRI, CT scan, or ultrasound) **
**Vascular disorders**	

Hypertension	
Systolic blood pressure (BP) 140–159 mm Hg or Diastolic BP 90–99 mm Hg	**Mandatory:** If recurrent or persistent (≥24 hrs), or if symptomatic increase by >20 mm Hg (diastolic) or to >140/90 mm Hg; monotherapy anti-hypertensive treatment indicated.<br>Maintain asciminib dose level, with regular monitoring of blood pressure.
Systolic BP ≥160 mm Hg or Diastolic BP ≥100 mm Hg	Medical intervention indicated: Initiate antihypertensive therapy (if not currently being treated with antihypertensive). If already taking anti- hypertensive drugs; intensive treatment with increased dose of existing anti-hypertensive/ more than one anti-hypertensive drugs than previously used<br>Hold asciminib dose until resolved to grade ≤2, then reduce dose 1 dose level
Common Terminology Criteria for Adverse Events (CTCAE) Grade 4	**Mandatory:** Permanently discontinue patient from treatment
**Gastrointestinal**	
Pancreatitis	
Grade 2 (radiologic findings for pancreatitis as per CTCAE v5.0, for increased enzymes please see table for asymptomatic amylase and/or lipase elevation)	**Mandatory:** If asymptomatic radiologic pancreatitis, hold treatment until recovery of the radiologic findings. If treatment delay is ≤21 days, then reduce dose □ 1 dose level. If treatment delay >21 days, discontinue treatment and keep monitoring with appropriate imaging (i.e., MRI, CT scan, or ultrasound) **
Grade ≥ 3	**Mandatory:** Permanently discontinue patient from treatment. Obtain appropriate imaging (i.e., MRI, CT scan, or ultrasound)
Diarrhea***	
Grade 1	**Recommendation:** Maintain dose level but initiate anti-diarrhea treatment
Grade 2	**Recommendation:** Hold dose until resolved to grade ≤ 1, then maintain dose level

	If diarrhea returns as grade ≥2, hold dose until resolved to grade ≤1, then reduce dose 1 dose level
Grade 3	**Recommendation:** Hold dose and discontinue patient from treatment
Grade 4	**Mandatory:** Permanently discontinue patient from treatment
**Skin and subcutaneous tissue disorders**	
Rash/photosensitivity	
Grade 1	**Recommendation:** Maintain dose level. Consider initiating appropriate<br>skin toxicity therapy (such as antihistamines, topical corticosteroids and low-dose systemic corticosteroids)
Grade 2	**Recommendation**: Maintain dose level, but initiate/intensify appropriate<br>skin toxicity therapy (such as antihistamines, topical corticosteroids and low-dose systemic corticosteroids)
Grade 3, despite skin toxicity therapy	**Recommendation:** Hold dose until resolved to grade ≤1, then:<br>If resolved in ≤7 days, reduce dose 1 dose level<br>If resolved in >7 days (despite appropriate skin toxicity therapy), discontinue patient from treatment
Grade 4, despite skin toxicity therapy	**Mandatory:** Permanently discontinue patient from treatment
**General disorders and administration site conditions**	
Fatigue/Asthenia	
Grade 1 or 2	**Recommendation:** Maintain dose level
Grade 3	**Recommendation:** Hold dose until resolved to grade ≤1, then:<br>If resolved in ≤7 days, maintain dose level<br>If resolved in >7 days, reduce dose 1 dose level
All dose modifications should be based on the worst preceding toxicity.<br>[a] Common Terminology Criteria for Adverse Events (CTCAE Version 5.0).	

[b] Core LFTs consist of ALT, AST, total bilirubin (fractionated [direct and indirect], if total bilirubin > 2.0 x ULN), and alkaline phosphatase (ALP) (fractionated [quantification of isoforms], if ALP > 2.0 x ULN).

[c] “Combined” defined as total bilirubin increase to the defined threshold concurrently with ALT/AST increase to the defined threshold.

If combined elevations of AST or ALT and total bilirubin do not meet the defined thresholds, please follow the instructions for isolated elevation of total bilirubin and isolated elevation of AST/ALT, and take a conservative action based on the degree of the elevations (e.g. discontinue treatment at the situation when hold dose is needed for one parameter and discontinue treatment is required for another parameter). After all elevations resolve to the defined thresholds that allow treatment re-initiation, re-start the treatment either at the same dose or at one dose lower if meeting a criterion for dose reduction.

[d] “Cholestasis” defined as ALP elevation (> 2.0 x ULN and R value < 2) in patients

Note: The R value is calculated by dividing the ALT by the ALP, using multiples of the ULN for both values. It denotes whether the relative pattern of ALT and/or ALP elevation is due to cholestatic (R ≤ 2), hepatocellular (R ≥ 5), or mixed (R >2 and < 5) liver injury.

* Note: If total bilirubin > 3.0 x ULN is due to the indirect (non-conjugated) component only, and hemolysis as the etiology has been ruled out as per institutional guidelines (e.g., review of peripheral blood smear and haptoglobin determination), then ↓ 1 dose level and continue treatment at the discretion of the investigator.

** Note: A CT scan or other imaging study to assess the pancreas, liver, and gallbladder should be performed within 1 week of the first occurrence of any grade ≥ 3 amylase and/or lipase elevation. If asymptomatic grade 3 elevations of lipase and/or amylase occur again at the reduced dose, patients will be discontinued permanently from study treatment. If asymptomatic grade 2 re-occur at reduce dose, closed monitoring of lipase and amylase will be required.

*** Note: Antidiarrheal medication is recommended at the first sign of abdominal cramping, loose stools, or overt diarrhea.

**** Note: An isolated bilirubin elevation is not typical for drug-induced liver injury. Bilirubin can be elevated either as part of a “Hy’s law” constellation with a preceding elevation of ALT/AST, or as part of a cholestatic reaction with simultaneous elevation of other cholestatic parameters (alkaline phosphatase, gamma-glutamyl transpeptidase). Isolated bilirubin can be seen in conjunction with drugs

that inhibit bilirubin conjugation or excretion, but both scenarios do not typically represent liver injury. Alternative causes of bilirubin elevation should therefore, be ruled out before basing dose modification decisions on bilirubin.

Propensity Scores Weighting analysis

This study was not powered to compare the two dosing regimens. Given the lack of balance in the main cohort baseline characteristics between the two treatment arms (40 mg BID and 80 mg QD), the propensity score weighting method was used to evaluate potential differences in MMR rates at Week 48 between the two dosing regimens used in the ASC4OPT study. The propensity score weighting method is a statistical technique that reduces confounding in observational studies by balancing treatment groups, allowing for the estimation of treatment effects and facilitating indirect comparisons when direct head-to-head comparisons are not feasible due to variations across studies or populations[4]. A similar analysis was conducted to compare MMR rates at Week 48 in ASC4OPT with those observed in the ASCEMBL study[3], taking into account baseline prognostic factors such as the reason of discontinuation of the prior TKI and the presence of *BCR::ABL1* mutations, given the differing inclusion criteria.

The propensity score e(X) is defined as the conditional probability of receiving a particular treatment given the vector of observed covariates X: e(X) = P(T = 1|X), where T denotes the treatment indicator (1 for treated, 0 for control). To create a pseudo-population in which the distribution of covariates is independent of the treatment assignment, weights are computed as follows:

- For treated individuals: w_i=1/e(Xi)
- For control individuals: w_i=1/(1-e(Xi))

These weights adjust for the imbalance in baseline covariates between treatment groups. When comparing multiple treatments across different datasets, propensity score weighting helps to adjust for baseline differences, making the treatment groups comparable.

The effective sample size (ESS) can be used to quantify how well the matching worked. It is given by,

$$ESS = \frac{(\sum_i w_i)^2}{\sum_i w_i^2}$$

References

1. Hochhaus A, Baccarani M, Silver RT, Schiffer C, Apperley JF, Cervantes F, Clark RE, Cortes JE, Deininger MW, Guilhot F, Hjorth-Hansen H, Hughes TP, Janssen JJWM, Kantarjian HM, Kim DW, Larson RA, Lipton JH, Mahon FX, Mayer J, Nicolini F, Niederwieser D, Pane F, Radich JP, Rea D, Richter J, Rosti G, Rousselot P, Saglio G, Saußele S, Soverini S, Steegmann JL, Turkina A, Zaritskey A, Hehlmann R. European LeukemiaNet 2020 recommendations for treating chronic myeloid leukemia. *Leukemia*. 2020;34(4):966-984.
2. Clark RE, Apperley JF, Copland M, Cicconi S. Additional chromosomal abnormalities at chronic myeloid leukemia diagnosis predict an increased risk of progression. *Blood Adv*. 2021;5(4):1102-1109.
3. Réa D, Mauro MJ, Boquimpani C, Minami Y, Lomaia E, Voloshin S, et al. A phase 3, open-label, randomized study of asciminib, a STAMP inhibitor, vs bosutinib in CML after 2 or more prior TKIs. *Blood*. 2021;138(21):2031-41.
4. Austin PC. An Introduction to Propensity Score Methods for Reducing the Effects of Confounding in Observational Studies. *Multivariate Behav Res.* 2011;46(3):399-424.

Supplementary Figures

Supplementary Figure 1. Study design

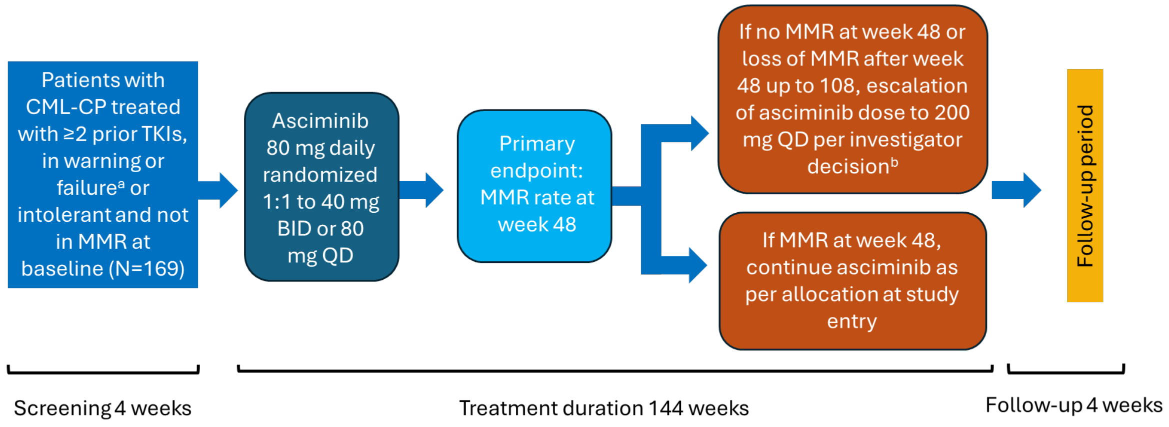

[a]Based on ELN 2020 recommendations. [b]In addition, there must not be any grade 3 or 4 toxicity while on therapy, or persistent grade 2 toxicity, possibly related to asciminib and unresponsive to optimal management.

Up to 30 additional patients in MMR at baseline and intolerant to most recent TKI treatment were enrolled (not included in the primary endpoint analysis). BID, twice daily; CML, chronic myeloid leukemia; CP, chronic phase; ELN, European LeukemiaNet; MMR, major molecular response (*BCR::ABL1*^{IS} ≤0.1%); QD, once daily; TKI, tyrosine kinase inhibitor.

Supplementary Figure 2. Sequence of prior TKIs, main cohort.

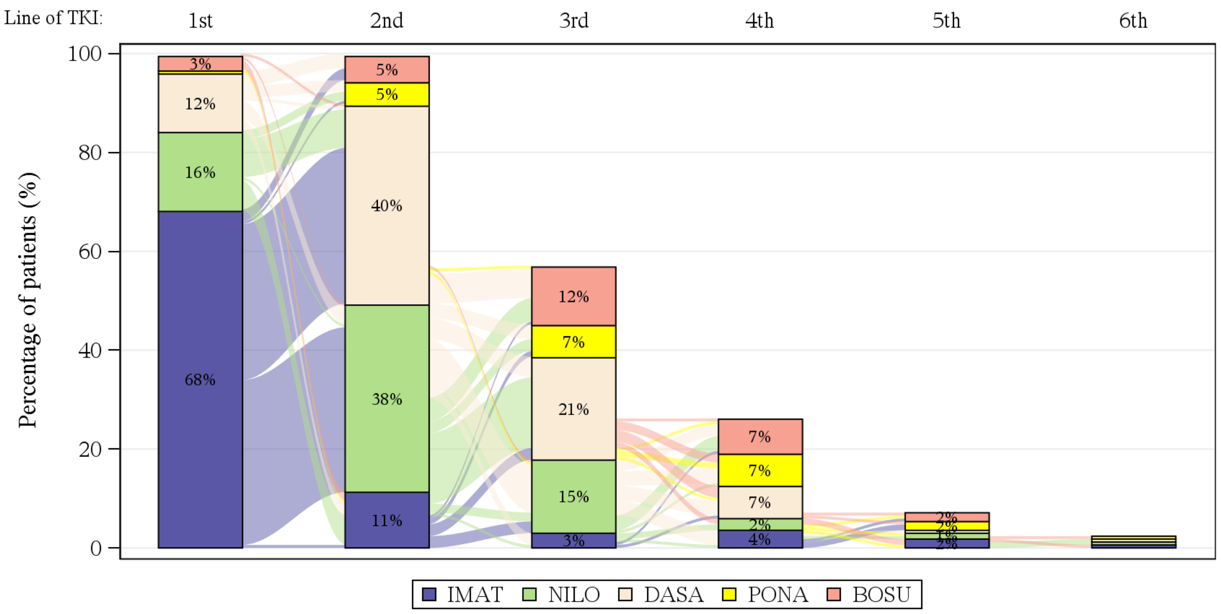

BOSU, bosutinib; DASA, dasatinib; IMA, imatinib; NILO, nilotinib; PONA, ponatinib; TKI, tyrosine kinase inhibitor.

Supplementary Figure 3. Kaplan-Meier estimate of duration of MMR among patients who achieved MMR, main cohort.

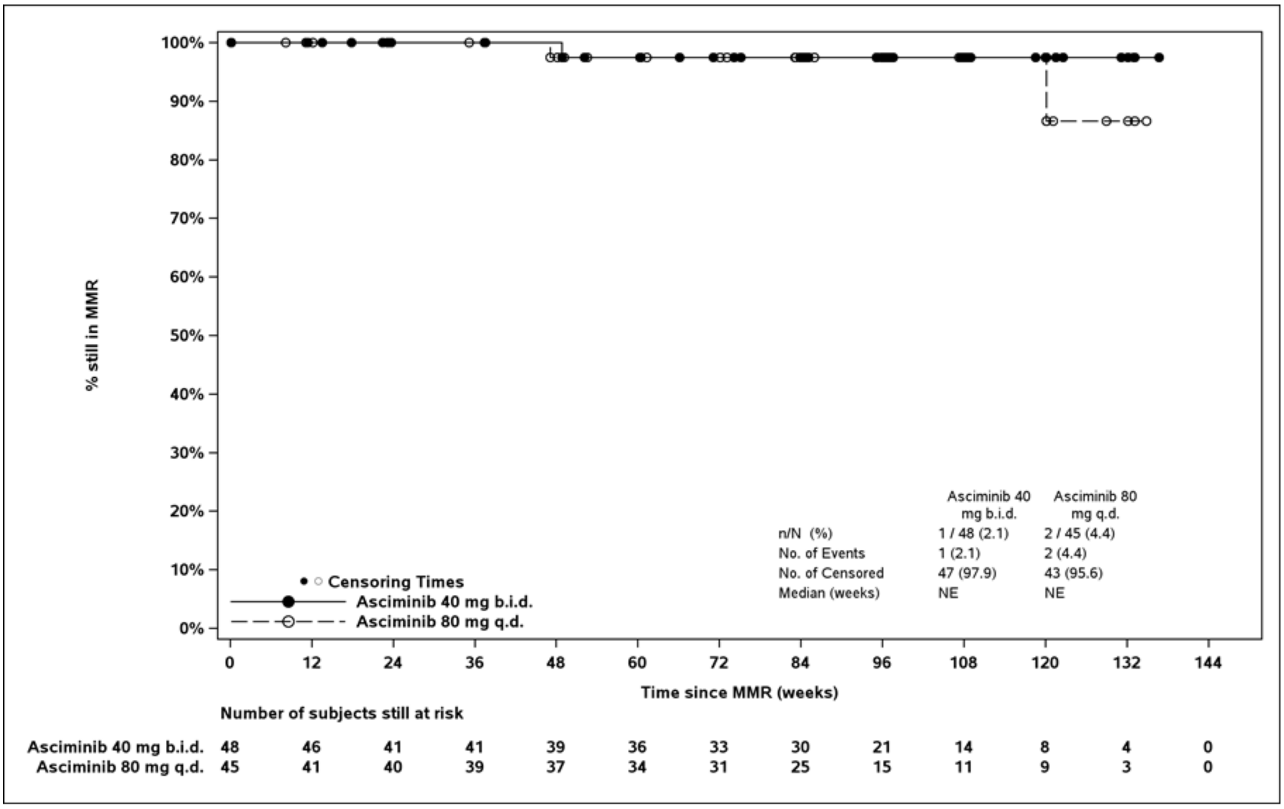

BID, twice daily; MMR, major molecular response; NE, not estimable; QD, once daily.

Supplementary Figure 4. MDASI-CML Symptom Total Score Derived (A) and Interference Total Score Derived (B) scores, main cohort.
Mean change from baseline (SD) is presented; patients with an evaluable baseline score and at least one evaluable post baseline score during the treatment period were included in the change from baseline analyses. Patients with partially completed questionnaires were included in the analysis. Score ranges from 0 to 10, with a higher score indicating greater symptom/interference severity. Number of patients remaining on treatment at each timepoint shown for different dosing schedules. Negative change shows improvement; positive change indicates deterioration.

A

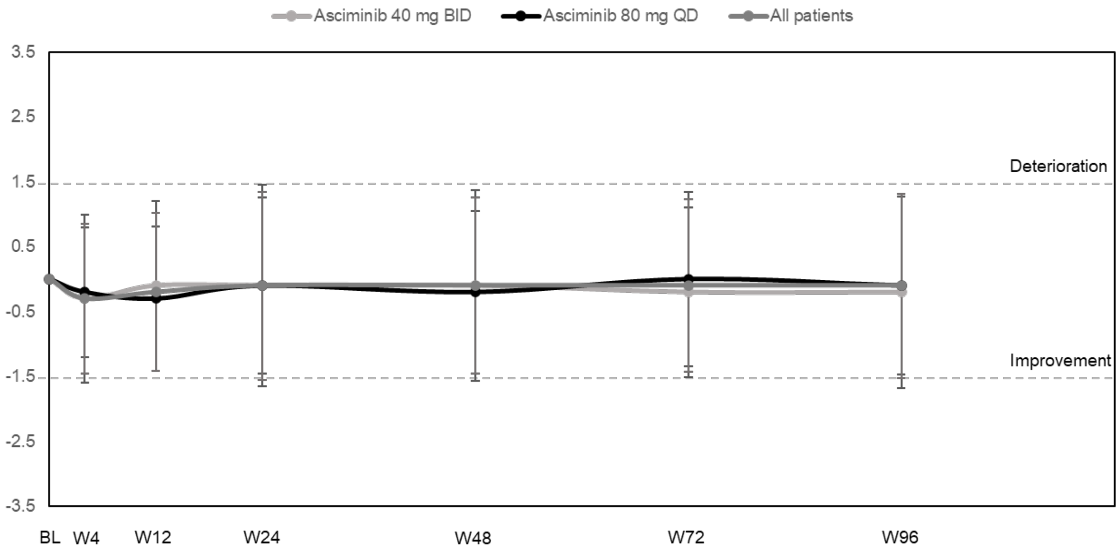

	BL	W4	W12	W24	W48	W72	W96
Asciminib 40 mg BID	84	83	77	74	72	66	63
Asciminib 80 mg QD	84	81	79	78	71	67	64
All patients	168	164	156	152	143	133	127

B

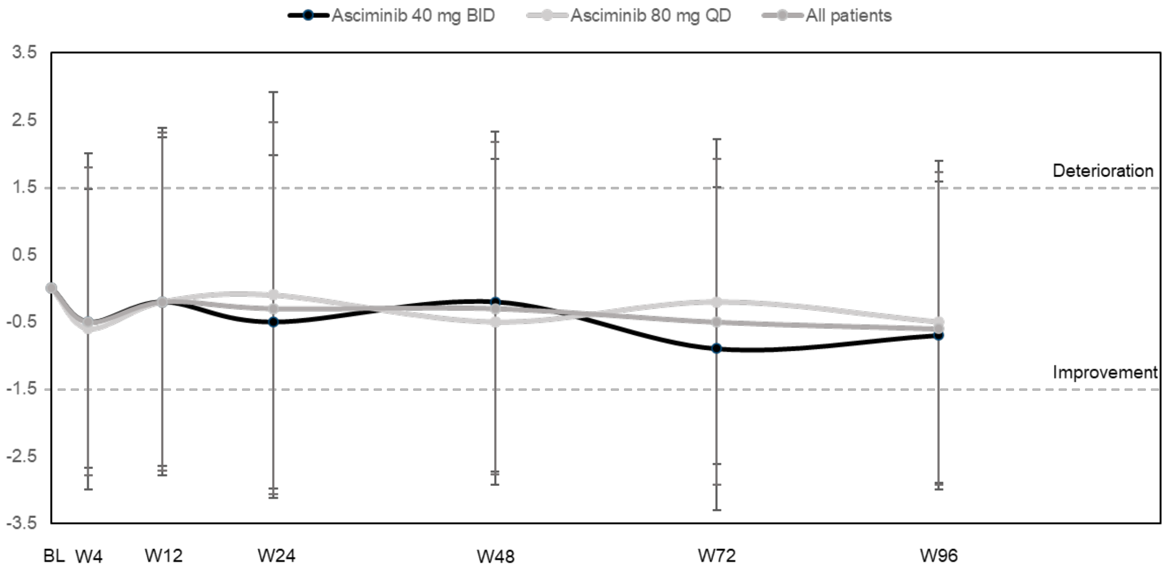

	BL	W4	W12	W24	W48	W72	W96
Asciminib 40 mg BID	84	81	76	73	69	64	63
Asciminib 80 mg QD	83	79	77	77	69	66	63
All patients	167	160	153	150	138	130	126

BID, twice daily; BL, baseline; MDASI-CML, MD Anderson Symptom Inventory-Chronic Myeloid Leukemia; QD, once daily; SD, standard deviation; W, week.

Supplementary Figure 5. MDASI-CML Symptom Total Score Derived (A) and Interference Total Score Derived (B) scores (exploratory cohort).
Mean change from baseline (SD) is presented; patients with an evaluable baseline score and at least one evaluable post baseline score during the treatment period were included in the change from baseline analyses. Patients with partially completed questionnaires were included in the analysis. Score ranges from 0 to 10, with a higher score indicating greater symptom/interference severity. Number of patients remaining on treatment at each timepoint shown for different dosing schedules. Negative change shows improvement; positive change indicates deterioration.

A

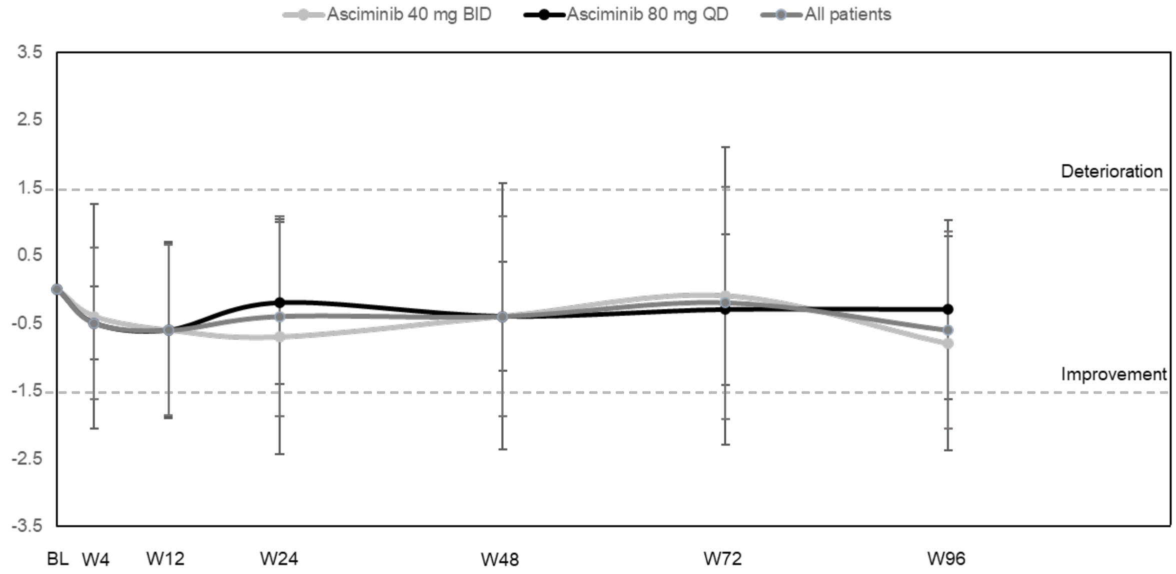

	BL	W4	W12	W24	W48	W72	W96
Asciminib 40 mg BID	13	10	12	13	13	13	12
Asciminib 80 mg QD	15	14	13	13	13	13	12
All patients	28	24	25	26	26	26	24

B

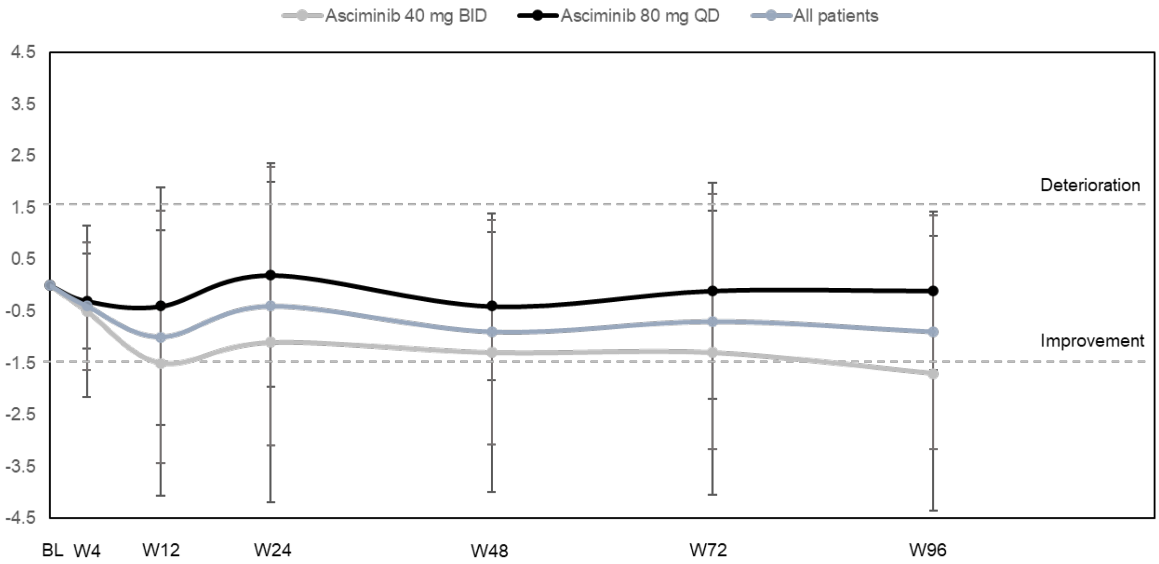

	BL	W4	W12	W24	W48	W72	W96
Asciminib 40 mg BID	13	9	12	13	13	13	12
Asciminib 80 mg QD	15	14	13	13	13	13	12
All patients	28	23	25	26	26	26	24

BID, twice daily; BL, baseline; MDASI-CML, MD Anderson Symptom Inventory-Chronic Myeloid Leukemia; QD, once daily; SD, standard deviation; W, week.

Supplementary Tables

Supplementary Table 1. Patient demographics and disease characteristics for the patients who received asciminib 200 mg QD, main cohort.

	Main cohort, patients who received asciminib 200 mg QD		
	**Asciminib 40 mg BID n=16**	**Asciminib 80 mg QD n=24**	**All patients N=40**
Median age, years (range)	46.5 (27–86)	49.5 (18–77)	48.0 (18–86)
Male, n (%)	9 (56.3)	16 (66.7)	25 (62.5)
Race, n (%)			
White	6 (37.5)	16 (66.7)	22 (55.0)
Black or African American	2 (12.5)	1 (4.2)	3 (7.5)
Asian	7 (43.8)	7 (29.2)	14 (35.0)
Unknown	1 (6.3)	0	1 (2.5)
Ethnicity, n (%)			
Hispanic or Latino	2 (12.5)	4 (16.7)	6 (15.0)
Not Hispanic or Latino	13 (81.3)	17 (70.8)	30 (75.0)
Not Reported	1 (6.3)	2 (8.3)	3 (7.5)
Unknown	0	1 (4.2)	1 (2.5)
Median time since initial diagnosis of CML, years (range)	4.1 (1.7–22.5)	3.7 (1.1–22.8)	3.7 (1.1–22.8)

BID, twice daily; CML, chronic myeloid leukemia; QD, once daily.

Supplementary Table 2. MMR rate at scheduled times (excluding patients with the T315 mutation detected after treatment start), main cohort.

	Asciminib 40 mg BID n=83		Asciminib 80 mg QD n=82		All patients N=165	
	n (%)	95% CI	n (%)	95% CI	n (%)	95% CI
**MMR**						
**at Week 12**	26 (31.3)	(21.59, 42.44)	20 (24.4)	(15.58, 35.12)	46 (27.9)	(21.19, 35.38)
**at Week 24**	30 (36.1)	(25.88, 47.43)	25 (30.5)	(20.80, 41.64)	55 (33.3)	(26.20, 41.08)
**at Week 36**	35 (42.2)	(31.40, 53.51)	30 (36.6)	(26.22, 47.95)	65 (39.4)	(31.89, 47.29)
**at Week 48**	36 (43.4)	(32.53, 54.71)	29 (35.4)	(25.12, 46.70)	65 (39.4)	(31.89, 47.29)
**at Week 60**	34 (41.0)	(30.28, 52.31)	28 (34.1)	(24.03, 45.45)	62 (37.6)	(30.17, 45.44)
**at Week 72**	36 (43.4)	(32.53, 54.71)	33 (40.2)	(29.56, 51.66)	69 (41.8)	(34.20, 49.74)
**at Week 84**	36 (43.4)	(32.53, 54.71)	35 (42.7)	(31.82, 54.09)	71 (43.0)	(35.36, 50.96)
**at Week 96**	38 (45.8)	(34.79, 57.08)	34 (41.5)	(30.68, 52.88)	72 (43.6)	(35.94, 51.56)

Pearson-Clopper 95% 2-sided CI for response rate. BID, twice daily; CI, confidence interval; MMR, major molecular response; QD, once daily.

Supplementary Table 3. MMR rate by scheduled times (excluding patients with the T315 mutation detected after treatment start), main cohort.

	Asciminib 40 mg BID n=83		Asciminib 80 mg QD n=82		All patients N=165	
	n (%)	95% CI	n (%)	95% CI	n (%)	95% CI
**MMR**						
**by Overall**	45 (54.2)	(42.92, 65.21)	39 (47.6)	(36.41, 58.89)	84 (50.9)	(43.02, 58.76)
**by Week 12**	26 (31.3)	(21.59, 42.44)	20 (24.4)	(15.58, 35.12)	46 (27.9)	(21.19, 35.38)
**by Week 24**	31 (37.3)	(26.97, 48.66)	26 (31.7)	(21.87, 42.92)	57 (34.5)	(27.33, 42.33)
**by Week 36**	37 (44.6)	(33.66, 55.90)	30 (36.6)	(26.22, 47.95)	67 (40.6)	(33.04, 48.52)
**by Week 48**	37 (44.6)	(33.66, 55.90)	32 (39.0)	(28.44, 50.43)	69 (41.8)	(34.20, 49.74)
**by Week 60**	38 (45.8)	(34.79, 57.08)	33 (40.2)	(29.56, 51.66)	71 (43.0)	(35.36, 50.96)
**by Week 72**	40 (48.2)	(37.08, 59.44)	37 (45.1)	(34.10, 56.51)	77 (46.7)	(38.87, 54.58)
**by Week 84**	42 (50.6)	(39.40, 61.76)	39 (47.6)	(36.41, 58.89)	81 (49.1)	(41.24, 56.98)
**by Week 96**	45 (54.2)	(42.92, 65.21)	39 (47.6)	(36.41, 58.89)	84 (50.9)	(43.02, 58.76)

Pearson-Clopper 95% 2-sided CI for response rate. BID, twice daily; CI, confidence interval; MMR, major molecular response; QD, once daily.

Supplementary Table 4. Comparison of baseline prognostic factors for propensity score weighting MMR rate analysis at Week 48, main cohort(excluding patients with the T315I mutation at baseline).

**Variables**		**ASCEMBL 40 mg BID N=155 n (%)**	**ASC4OPT 40 mg BID n=83 n (%)**	**ASC4OPT 80 mg QD n=82 n (%)**
Age group	<65 years	126 (81.3)	62 (74.7)	57 (69.5)
	≥65 years	29 (18.7)	21 (25.3)	25 (30.5)
Sex	Female	75 (48.4)	37 (44.6)	26 (31.7)
	Male	80 (51.6)	46 (55.4)	56 (68.3)
Reason for discontinuation of last prior TKI	Resistance	94 (60.6)	42 (50.6)	45 (54.9)
	Intolerance	58 (37.4)	19 (22.9)	20 (24.4)
	Unknown/Other	3 (1.9)	22 (26.5)	17 (20.7)
Response at baseline	*BCR::ABL1*^{IS} ≤1%	15 (9.7)	30 (36.1)	26 (31.7)
	BCR::ABL1^{IS} >1%	140 (90.3)	53 (63.9)	56 (68.3)
Number of prior TKI therapies	2	88 (56.8)	46 (55.4)	41 (50)
	≥3	67 (43.2)	37 (44.6)	41 (50)
Mutation	Mutant	17 (11)	5 (6)	11 (13.4)
	Unmutated	125 (80.6)	66 (79.5)	61 (74.4)
	Unknown	13 (8.4)	12 (14.5)	10 (12.2)

BID, twice daily; MMR, major molecular response; QD, once daily; TKI, tyrosine kinase inhibitor.

Supplementary Table 5. Analysis of MMR rates on asciminib 40 mg BID vs 80 mg QD in ASC4OPT and vs 40 mg BID in ASCEMBL using propensity score weighting, main cohort (excluding patients with the T315I mutation at baseline).

Study	N	MMR rate (%)	Difference	95% CI	P-value
**Asciminib 40 mg BID vs 80 mg QD in ASC4OPT**					
ASC4OPT 80 mg QD	82	35.37	2.38	(-13.09, 17.85)	0.763[b]
ASC4OPT 40 mg BID adjusted	68[a]	37.75			
ASC4OPT 40 mg BID unadjusted	83	43.37			
**Asciminib 40 mg BID in ASC4OPT vs 40 mg BID in ASCEMBL**					
ASC4OPT 40 mg BID	61[c]	40.98	-2.37	(-19.23, 14.48)	0.783[b]
ASCEMBL 40 mg BID adjusted	69[a]	38.61			
ASCEMBL 40 mg BID unadjusted	152[c]	30.26			
**Asciminib 40 mg BID in ASC4OPT vs 80 mg QD in ASCEMBL**					
ASC4OPT 80 mg QD	65[c]	33.85	1.03	(-14.28, 16.33)	0.895[b]
ASCEMBL 40 mg BID adjusted	86[a]	34.87			
ASCEMBL 40 mg BID unadjusted	152[c]	30.26			

[a]Effective sample size = square of the summed weights / sum of squared weights. [b]Computed only for ad-hoc analysis. [c]Including only patients in ASC4OPT with either failure or intolerance cited as reason of discontinuation of prior TKI, to match the population criteria of ASCEMBL with regard to ELN guidelines. BID, twice daily; CI, confidence interval; ELN, European LeukemiaNet; MMR, major molecular response; QD, once daily; TKI, tyrosine kinase inhibitor.

Supplementary Table 6. MR^4 rate at scheduled times, main cohort (excluding patients with the T315I mutation at baseline).

	Asciminib 40 mg BID n=83		Asciminib 80 mg QD n=82		All patients N=165	
	n (%)	95% CI	n (%)	95% CI	n (%)	95% CI
**MR^4**						
**at Week 12**	8 (9.6)	(4.25, 18.11)	3 (3.7)	(0.76, 10.32)	11 (6.7)	(3.37, 11.62)
**at Week 24**	19 (22.9)	(14.38, 33.42)	9 (11.0)	(5.14, 19.82)	28 (17.0)	(11.58, 23.58)
**at Week 36**	17 (20.5)	(12.41, 30.76)	10 (12.2)	(6.01, 21.29)	27 (16.4)	(11.07, 22.91)
**at Week 48**	17 (20.5)	(12.41, 30.76)	11 (13.4)	(6.89, 22.74)	28 (17.0)	(11.58, 23.58)
**at Week 60**	19 (22.9)	(14.38, 33.42)	11 (13.4)	(6.89, 22.74)	30 (18.2)	(12.62, 24.93)
**at Week 72**	19 (22.9)	(14.38, 33.42)	12 (14.6)	(7.80, 24.17)	31 (18.8)	(13.14, 25.60)
**at Week 84**	19 (22.9)	(14.38, 33.42)	12 (14.6)	(7.80, 24.17)	31 (18.8)	(13.14, 25.60)
**at Week 96**	18 (21.7)	(13.39, 32.09)	10 (12.2)	(6.01, 21.29)	28 (17.0)	(11.58, 23.58)

Pearson-Clopper 95% 2-sided CI for response rate. BID, twice daily; CI, confidence interval; MR, molecular response; QD, once daily.

Supplementary Table 7. MR^4 rate by scheduled times, main cohort (excluding patients with the T315I mutation at baseline).

	Asciminib 40 mg BID n=83		Asciminib 80 mg QD n=82		All patients N=165	
	n (%)	95% CI	n (%)	95% CI	n (%)	95% CI
**MR^4**						
**by Overall**	22 (26.5)	(17.42, 37.34)	14 (17.1)	(9.66, 26.98)	36 (21.8)	(15.77, 28.90)
**by Week 12**	8 (9.6)	(4.25, 18.11)	3 (3.7)	(0.76, 10.32)	11 (6.7)	(3.37, 11.62)
**by Week 24**	19 (22.9)	(14.38, 33.42)	9 (11.0)	(5.14, 19.82)	28 (17.0)	(11.58, 23.58)
**by Week 36**	19 (22.9)	(14.38, 33.42)	11 (13.4)	(6.89, 22.74)	30 (18.2)	(12.62, 24.93)
**by Week 48**	19 (22.9)	(14.38, 33.42)	12 (14.6)	(7.80, 24.17)	31 (18.8)	(13.14, 25.60)
**by Week 60**	21 (25.3)	(16.39, 36.04)	13 (15.9)	(8.72, 25.58)	34 (20.6)	(14.71, 27.59)
**by Week 72**	22 (26.5)	(17.42, 37.34)	14 (17.1)	(9.66, 26.98)	36 (21.8)	(15.77, 28.90)
**by Week 84**	22 (26.5)	(17.42, 37.34)	14 (17.1)	(9.66, 26.98)	36 (21.8)	(15.77, 28.90)
**by Week 96**	22 (26.5)	(17.42, 37.34)	14 (17.1)	(9.66, 26.98)	36 (21.8)	(15.77, 28.90)

Pearson-Clopper 95% 2-sided CI for response rate. BID, twice daily; CI, confidence interval; MR, molecular response; QD, once daily.

Supplementary Table 8. $MR^{4.5}$ rate at scheduled times, main cohort (excluding patients with the T351I mutation at baseline).

	Asciminib 40 mg BID n=83		Asciminib 80 mg QD n=82		All patients N=165	
	n (%)	95% CI	n (%)	95% CI	n (%)	95% CI
**$MR^{4.5}$**						
**at Week 12**	1 (1.2)	(0.03, 6.53)	1 (1.2)	(0.03, 6.61)	2 (1.2)	(0.15, 4.31)
**at Week 24**	10 (12.0)	(5.93, 21.04)	4 (4.9)	(1.34, 12.02)	14 (8.5)	(4.72, 13.83)
**at Week 36**	12 (14.5)	(7.70, 23.89)	6 (7.3)	(2.73, 15.25)	18 (10.9)	(6.59, 16.69)
**at Week 48**	10 (12.0)	(5.93, 21.04)	7 (8.5)	(3.50, 16.80)	17 (10.3)	(6.12, 15.98)
**at Week 60**	12 (14.5)	(7.70, 23.89)	7 (8.5)	(3.50, 16.80)	19 (11.5)	(7.08, 17.40)
**at Week 72**	14 (16.9)	(9.54, 26.68)	6 (7.3)	(2.73, 15.25)	20 (12.1)	(7.56, 18.10)
**at Week 84**	14 (16.9)	(9.54, 26.68)	6 (7.3)	(2.73, 15.25)	20 (12.1)	(7.56, 18.10)
**at Week 96**	11 (13.3)	(6.81, 22.48)	7 (8.5)	(3.50, 16.80)	18 (10.9)	(6.59, 16.69)

Pearson-Clopper 95% 2-sided CI for response rate. BID, twice daily; CI, confidence interval; MR, molecular response; QD, once daily.

Supplementary Table 9. $MR^{4.5}$ rate by scheduled times, main cohort (excluding patients with the T351I mutation at baseline).

	Asciminib 40 mg BID n=83		Asciminib 80 mg QD n=82		All patients N=165	
	n (%)	95% CI	n (%)	95% CI	n (%)	95% CI
**$MR^{4.5}$**						
**by Overall**	18 (21.7)	(13.39, 32.09)	10 (12.2)	(6.01, 21.29)	28 (17.0)	(11.58, 23.58)
**by Week 12**	1 (1.2)	(0.03, 6.53)	1 (1.2)	(0.03, 6.61)	2 (1.2)	(0.15, 4.31)
**by Week 24**	10 (12.0)	(5.93, 21.04)	4 (4.9)	(1.34, 12.02)	14 (8.5)	(4.72, 13.83)
**by Week 36**	14 (16.9)	(9.54, 26.68)	6 (7.3)	(2.73, 15.25)	20 (12.1)	(7.56, 18.10)
**by Week 48**	15 (18.1)	(10.48, 28.05)	7 (8.5)	(3.50, 16.80)	22 (13.3)	(8.55, 19.49)
**by Week 60**	15 (18.1)	(10.48, 28.05)	8 (9.8)	(4.31, 18.32)	23 (13.9)	(9.05, 20.18)
**by Week 72**	18 (21.7)	(13.39, 32.09)	9 (11.0)	(5.14, 19.82)	27 (16.4)	(11.07, 22.91)
**by Week 84**	18 (21.7)	(13.39, 32.09)	9 (11.0)	(5.14, 19.82)	27 (16.4)	(11.07, 22.91)
**by Week 96**	18 (21.7)	(13.39, 32.09)	10 (12.2)	(6.01, 21.29)	28 (17.0)	(11.58, 23.58)

Pearson-Clopper 95% 2-sided CI for response rate. BID, twice daily; CI, confidence interval; MR, molecular response; QD, once daily.

Supplementary Table 10. *BCR::ABL1*^{IS} ≤1% rate at scheduled times, main cohort (excluding patients with the T315I mutation at baseline).

	Asciminib 40 mg BID n=83		Asciminib 80 mg QD n=82		All patients N=165	
	n (%)	95% CI	n (%)	95% CI	n (%)	95% CI
***BCR::ABL1*^{IS} ≤1%**						
**at Baseline**	30 (36.1)	(25.88, 47.43)	26 (31.7)	(21.87, 42.92)	56 (33.9)	(26.76, 41.71)
**at Week 12**	52 (62.7)	(51.34, 73.03)	45 (54.9)	(43.49, 65.90)	97 (58.8)	(50.87, 66.38)
**at Week 24**	55 (66.3)	(55.05, 76.28)	50 (61.0)	(49.57, 71.56)	105 (63.6)	(55.80, 70.97)
**at Week 36**	54 (65.1)	(53.81, 75.20)	54 (65.9)	(54.55, 75.97)	108 (65.5)	(57.67, 72.67)
**at Week 48**	55 (66.3)	(55.05, 76.28)	50 (61.0)	(49.57, 71.56)	105 (63.6)	(55.80, 70.97)
**at Week 60**	54 (65.1)	(53.81, 75.20)	52 (63.4)	(52.05, 73.78)	106 (64.2)	(56.42, 71.54)
**at Week 72**	55 (66.3)	(55.05, 76.28)	53 (64.6)	(53.30, 74.88)	108 (65.5)	(57.67, 72.67)
**at Week 84**	54 (65.1)	(53.81, 75.20)	54 (65.9)	(54.55, 75.97)	108 (65.5)	(57.67, 72.67)
**at Week 96**	52 (62.7)	(51.34, 73.03)	55 (67.1)	(55.81, 77.06)	107 (64.8)	(57.04, 72.11)

Pearson-Clopper 95% 2-sided CI for response rate. BID, twice daily; CI, confidence interval; IS, international scale; QD, once daily.

Supplementary Table 11. *BCR::ABL1*^{IS} ≤1% rate by scheduled times, main cohort (excluding patients with the T315I mutation at baseline).

	Asciminib 40 mg BID n=83		Asciminib 80 mg QD n=82		All patients N=165	
	n (%)	95% CI	n (%)	95% CI	n (%)	95% CI
***BCR::ABL1*^{IS} ≤1%**						
**by Overall**	58 (69.9)	(58.82, 79.47)	61 (74.4)	(63.56, 83.40)	119 (72.1)	(64.62, 78.81)
**by Week 12**	52 (62.7)	(51.34, 73.03)	45 (54.9)	(43.49, 65.90)	97 (58.8)	(50.87, 66.38)
**by Week 24**	56 (67.5)	(56.30, 77.35)	52 (63.4)	(52.05, 73.78)	108 (65.5)	(57.67, 72.67)
**by Week 36**	57 (68.7)	(57.56, 78.41)	57 (69.5)	(58.36, 79.20)	114 (69.1)	(61.44, 76.04)
**by Week 48**	58 (69.9)	(58.82, 79.47)	57 (69.5)	(58.36, 79.20)	115 (69.7)	(62.07, 76.60)
**by Week 60**	58 (69.9)	(58.82, 79.47)	58 (70.7)	(59.65, 80.26)	116 (70.3)	(62.71, 77.15)
**by Week 72**	58 (69.9)	(58.82, 79.47)	59 (72.0)	(60.94, 81.32)	117 (70.9)	(63.34, 77.71)
**by Week 84**	58 (69.9)	(58.82, 79.47)	60 (73.2)	(62.24, 82.36)	118 (71.5)	(63.98, 78.26)
**by Week 96**	58 (69.9)	(58.82, 79.47)	61 (74.4)	(63.56, 83.40)	119 (72.1)	(64.62, 78.81)

Pearson-Clopper 95% 2-sided CI for response rate. BID, twice daily; CI, confidence interval; IS, international scale; QD, once daily.

Supplementary Table 12. MR^4 rate at scheduled times, exploratory cohort.

	Asciminib 40 mg BID n=14		Asciminib 80 mg QD n=16		All patients N=30	
	n (%)	95% CI	n (%)	95% CI	n (%)	95% CI
**MR^4**						
**at Baseline**	3 (21.4)	(4.66, 50.80)	5 (31.3)	(11.02, 58.66)	8 (26.7)	(12.28, 45.89)
**at Week 12**	6 (42.9)	(17.66, 71.14)	6 (37.5)	(15.20, 64.57)	12 (40.0)	(22.66, 59.40)
**at Week 24**	6 (42.9)	(17.66, 71.14)	6 (37.5)	(15.20, 64.57)	12 (40.0)	(22.66, 59.40)
**at Week 36**	6 (42.9)	(17.66, 71.14)	6 (37.5)	(15.20, 64.57)	12 (40.0)	(22.66, 59.40)
**at Week 48**	7 (50.0)	(23.04, 76.96)	8 (50.0)	(24.65, 75.35)	15 (50.0)	(31.30, 68.70)
**at Week 60**	8 (57.1)	(28.86, 82.34)	8 (50.0)	(24.65, 75.35)	16 (53.3)	(34.33, 71.66)
**at Week 72**	8 (57.1)	(28.86, 82.34)	8 (50.0)	(24.65, 75.35)	16 (53.3)	(34.33, 71.66)
**at Week 84**	6 (42.9)	(17.66, 71.14)	8 (50.0)	(24.65, 75.35)	14 (46.7)	(28.34, 65.67)
**at Week 96**	7 (50.0)	(23.04, 76.96)	9 (56.3)	(29.88, 80.25)	16 (53.3)	(34.33, 71.66)

Pearson-Clopper 95% 2-sided CI for response rate. BID, twice daily; CI, confidence interval; MR, molecular response; QD, once daily.

Supplementary Table 13. $MR^{4.5}$ rate at scheduled times, exploratory cohort.

	Asciminib 40 mg BID n=14		Asciminib 80 mg QD n=16		All patients N=30	
	n (%)	95% CI	n (%)	95% CI	n (%)	95% CI
**$MR^{4.5}$**						
**at Baseline**	2 (14.3)	(1.78, 42.81)	2 (12.5)	(1.55, 38.35)	4 (13.3)	(3.76, 30.72)
**at Week 12**	4 (28.6)	(8.39, 58.10)	3 (18.8)	(4.05, 45.65)	7 (23.3)	(9.93, 42.28)
**at Week 24**	5 (35.7)	(12.76, 64.86)	4 (25.0)	(7.27, 52.38)	9 (30.0)	(14.73, 49.40)
**at Week 36**	5 (35.7)	(12.76, 64.86)	4 (25.0)	(7.27, 52.38)	9 (30.0)	(14.73, 49.40)
**at Week 48**	5 (35.7)	(12.76, 64.86)	3 (18.8)	(4.05, 45.65)	8 (26.7)	(12.28, 45.89)
**at Week 60**	5 (35.7)	(12.76, 64.86)	4 (25.0)	(7.27, 52.38)	9 (30.0)	(14.73, 49.40)
**at Week 72**	6 (42.9)	(17.66, 71.14)	4 (25.0)	(7.27, 52.38)	10 (33.3)	(17.29, 52.81)
**at Week 84**	5 (35.7)	(12.76, 64.86)	4 (25.0)	(7.27, 52.38)	9 (30.0)	(14.73, 49.40)
**at Week 96**	5 (35.7)	(12.76, 64.86)	4 (25.0)	(7.27, 52.38)	9 (30.0)	(14.73, 49.40)

Pearson-Clopper 95% 2-sided CI for response rate. BID, twice daily; CI, confidence interval; MR, molecular response; QD, once daily.

Supplementary Table 14. Overview of adverse events, main cohort.

Preferred term	Asciminib 40 mg BID n=84		Asciminib 80 mg QD n=84		All patients N=168	
	All grades n (%)	Grade ≥3 n (%)	All grades n (%)	Grade ≥3 n (%)	All grades n (%)	Grade ≥3 n (%)
Adverse events	79 (94.0)	27 (32.1)	79 (94.0)	36 (42.9)	158 (94.0)	63 (37.5)
AEs leading to discontinuation	7 (8.3)	3 (3.6)	5 (6.0)	4 (4.8)	12 (7.1)	7 (4.2)
AEs leading to dose adjustment/interruption	29 (34.5)	20 (23.8)	28 (33.3)	22 (26.2)	57 (33.9)	42 (25.0)

Numbers (n) represent counts of patients. A patient with multiple severity grades for an AE was only counted under the maximum grade. AEs occurring during treatment or within 30 days of last study medication are summarized. MedDRA version 26.1, CTCAE version 5.0. AE, adverse event; BID, twice daily; CTCAE, Common Terminology Criteria for Adverse Events; MedDRA, Medical Dictionary for Regulatory Activities; QD, once daily.

Supplementary Table 15. Overview of adverse events, exploratory cohort.

Preferred term	Asciminib 40 mg BID N=14		Asciminib 80 mg QD N=16		All patients N=30	
	All grades n (%)	Grade ≥3 n (%)	All grades n (%)	Grade ≥3 n (%)	All grades n (%)	Grade ≥3 n (%)
Adverse events	13 (92.9)	6 (42.9)	16 (100)	8 (50.0)	29 (96.7)	14 (46.7)
AEs leading to discontinuation	0	0	2 (12.5)	2 (12.5)	2 (6.7)	2 (6.7)
AEs leading to dose adjustment/interruption	5 (35.7)	4 (28.6)	4 (25.0)	2 (12.5)	9 (30.0)	6 (20.0)

Numbers (n) represent counts of patients. A patient with multiple severity grades for an AE is only counted under the maximum grade. AEs occurring during treatment or within 30 days of last study medication are summarized. MedDRA version 26.1, CTCAE version 5.0. AE, adverse event; BID, twice daily; CTCAE, Common Terminology Criteria for Adverse Events; MedDRA, Medical Dictionary for Regulatory Activities; QD, once daily.

Supplementary Table 16. Mutations detected at baseline by NGS, main cohort.

Patient	Mutations						Dose escalation	Current disease/survival status
	Baseline		Week 48		EOT			
	Mutation	VAF (%)	Mutation	VAF (%)	Mutation	VAF (%)		
1	F317L	99.05	F317L	100	NA	NA	Yes (at Week 60)	Ongoing
2	M244V	94.58	NA	NA	ND	ND	NA	Discontinued treatment
3	E453K	10.14	NA	NA	E453K, A433T	1.24, 8.22	NA	Discontinued treatment
4	M244V	99.7	NA[a]	NA	ND	ND	NA	Discontinued treatment
5	E255K	99.94	F359V	100	F359V	99.8	Yes (after Week 48)	Discontinued treatment
6	V299L	99.4	NA	NA	NA	NA	NA	Discontinued treatment due to an AE
7	T315I	78.72	NA	NA	T315I	97.5	NA	Discontinued treatment
8	T315I	99.85	NA	NA	ND	ND	NA	Discontinued treatment
9	Y253H	23.46	ND	ND	NA	NA	Yes (at Week 84)	Ongoing
10	E459K	8.2	NA	NA	E459K	8.5	NA	Discontinued treatment due to an AE
11	F486S, E255K	99.68, 99.79	NA	NA	ND	ND	NA	Discontinued treatment due to an AE
12	Y253H	98.87	ND	ND	NA	NA	No	Ongoing
13	F359I	86.54	NA	NA	F359I	99.7	NA	Discontinued treatment
14	E255K	36.72	M244V	100	M244V	98.4	NA	Discontinued treatment
15	E255V	99.85	NA	NA	NA	NA	NA	Ongoing
16	T315I	14.98	NA	NA	ND	ND	NA	Discontinued treatment
17	G250E	99.91	NA	NA	NA	NA	NA	Ongoing
18	M244V, G250E	27.33, 99.91	G250E	100	M244V, G250E	1.59, 99.91	Yes (at Week 72)	Discontinued treatment
19	Y253H	99.01	ND	ND	NA	NA	Yes (at Week 84)	Ongoing
20	Y253H	99.72	NA	NA	NA	NA	NA	Ongoing
21	Y253H	99.87	NA	NA	NA	NA	NA	Ongoing
22	G250E	99.83	NA	NA	NA	NA	NA	Ongoing
23	T315I	44.22	NA	NA	T315I, F459V, Q252H	43.07, 2.53, 1.2	NA	Discontinued treatment
24	M244V	75.75	NA	NA	M244V	99.2	NA	Discontinued treatment
25	Y253H	90.74	NA	NA	NA	NA	NA	Ongoing

[a] M244V mutation detected at Week 60 (VAF 99%). AE, adverse event; EOT, end of treatment; NA, not applicable; ND, not detected; NGS, next-generation sequencing; VAF, variant allele frequency.

Supplementary Table 17. Post-baseline mutations detected by NGS, main cohort.

Patients	Post-baseline mutations				Dose escalation	Current disease/survival status
	Week 48		EOT			
	Mutation	VAF (%)	Mutation	VAF (%)		
1	NA	NA	T315I	3.62	No	Discontinued treatment
2	M244V	ND	M244V	83.9	No	Discontinued treatment
3	P465A	ND	P465A	99.3	No	Discontinued treatment
4	NA	NA	M244V	ND	No	Ongoing
5	NA	NA	A68P<br>F317L	18.7<br>49.7	No	Discontinued treatment
6	NA	NA	K294E	ND	No	Ongoing
7	E453K	10.1	E453K<br>A433T	8.2<br>1.2	No	Discontinued treatment
8	M244V	ND	M244V	98.5	No	Discontinued treatment
9	F359V	ND	F359V	99.8	Yes	Discontinued treatment

EOT, end of treatment; NA, not applicable; ND, not determined; NGS, next-generation sequencing; VAF, variant allele frequency.
